# Reduced Proficiency in Homologous Recombination Underlies the High Sensitivity of Embryonal Carcinoma Testicular Germ Cell Tumors to Cisplatin and Poly (ADP-Ribose) Polymerase Inhibition

**DOI:** 10.1371/journal.pone.0051563

**Published:** 2012-12-12

**Authors:** Francesca Cavallo, Grazia Graziani, Cristina Antinozzi, Darren R. Feldman, Jane Houldsworth, George J. Bosl, Raju S. K. Chaganti, Mary Ellen Moynahan, Maria Jasin, Marco Barchi

**Affiliations:** 1 Department of Biomedicine and Prevention, Section of Anatomy, University of Rome Tor Vergata, Rome, Italy; 2 Developmental Biology Program, Memorial Sloan-Kettering Cancer Center, New York, New York, United States of America; 3 Department of System Medicine, University of Rome Tor Vergata, Rome, Italy; 4 Department of Medicine, Memorial Sloan-Kettering Cancer Center, New York, New York, United States of America; 5 Cell Biology Program, Memorial Sloan-Kettering Cancer Center, New York, New York, United States of America; National Cancer Institute, United States of America

## Abstract

Testicular Germ Cell Tumors (TGCT) and patient-derived cell lines are extremely sensitive to cisplatin and other interstrand cross-link (ICL) inducing agents. Nevertheless, a subset of TGCTs are either innately resistant or acquire resistance to cisplatin during treatment. Understanding the mechanisms underlying TGCT sensitivity/resistance to cisplatin as well as the identification of novel strategies to target cisplatin-resistant TGCTs have major clinical implications. Herein, we have examined the proficiency of five embryonal carcinoma (EC) cell lines to repair cisplatin-induced ICLs. Using γH2AX staining as a marker of double strand break formation, we found that EC cell lines were either incapable of or had a reduced ability to repair ICL-induced damage. The defect correlated with reduced Homologous Recombination (HR) repair, as demonstrated by the reduction of RAD51 foci formation and by direct evaluation of HR efficiency using a GFP-reporter substrate. HR-defective tumors cells are known to be sensitive to the treatment with poly(ADP-ribose) polymerase (PARP) inhibitor. In line with this observation, we found that EC cell lines were also sensitive to PARP inhibitor monotherapy. The magnitude of sensitivity correlated with HR-repair reduced proficiency and with the expression levels and activity of PARP1 protein. In addition, we found that PARP inhibition strongly enhanced the response of the most resistant EC cells to cisplatin, by reducing their ability to overcome the damage. These results point to a reduced proficiency of HR repair as a source of sensitivity of ECs to ICL-inducing agents and PARP inhibitor monotherapy, and suggest that pharmacological inhibition of PARP can be exploited to target the stem cell component of the TGCTs (namely ECs) and to enhance the sensitivity of cisplatin-resistant TGCTs to standard treatments.

## Introduction

Testicular germ cell tumors (TGCTs) develop from pre-malignant intratubular germ cell neoplasia and are histologically distinguished in seminomas and nonseminomas. The latter include yolk sac tumors and choriocarcinomas that represent extraembryonic cell differentiation, teratomas that represent somatic cell differentiation, and embryonal carcinomas (ECs) [1]. ECs are the malignant counterparts to embryonic stem cells and are considered the pluripotent stem cell component of nonseminomatous TGCTs [2]. As such, they are postulated to be the precursor of the other nonseminomatous histological entities.

TGCTs are highly curable with approximately 95% of newly diagnosed patients in 2012 expected to be rendered long-term disease-free. This includes more than 70% of patients with advanced (metastatic) disease, distinguishing TGCTs from most other solid tumors. Underlying this unique curability is the exquisite sensitivity of TGCTs to cisplatin-based chemotherapy [3], [4]. However, a subset of TGCTs are either innately resistant (rare) or acquire resistance to cisplatin-based therapy (more common) during cisplatin treatment. Although high-dose chemotherapy and surgery can overcome cisplatin-resistance in some cases, the majority of patients with platinum-resistant TGCT will ultimately die of disease. Tumor recurrence is also a major concern in TGCT patients, and it usually occurs within 2 years after initial treatment. Multiple studies have identified the presence of vascular invasion and the concomitant presence of EC-dominant tumors, as additive-risk factors for tumor recurrence in stage 1 non-seminoma TGCTs [5], [6]. This is likely, because the invading element is commonly, the EC component [7]. Therefore, the development of new therapeutic strategies to target ECs, and platinum-resistant TGCTs represents a clinical priority.

The underlying biological mechanism(s) responsible for the cisplatin sensitivity/resistance of TGCTs remains unknown. Several reports indicate that one mechanism for the unique sensitivity of TGCTs to DNA damaging agents is their exceptional apoptotic response [8]. Another attractive hypothesis is that TGCTs display a reduced capacity to repair cisplatin-induced DNA damage [1], [9], [10].

Cisplatin causes multiple types of DNA damage, such as mono-adducts, intrastrand crosslinks, DNA-protein crosslinks and interstrand crosslinks (ICLs). Despite comprising only a small fraction of cisplatin-induced DNA damage, ICLs are considered the most cytotoxic and genotoxic lesions caused by the drug. Indeed, ICLs covalently link the two strands of the double helix, causing a block of transcription and DNA replication [11]. DNA repair mechanisms play a pivotal role in cellular tolerance to cisplatin by bypassing or removing ICLs. The latter requires several classes of proteins including the nucleotide excision repair (NER) proteins XPF-ERCC1, translesion DNA-polymerases, Fanconi anemia gene products [12], [13], [14], and homologous recombination repair (HR) factors [15]. Double strand breaks (DSBs) near the ICL-site were observed as a pivotal intermediate in ICL repair and their formation only occurred after passage through S-phase [16]. Indeed, the prevailing model for ICL-repair suggests that following collision of the DNA replication fork with an ICL lesion, removal of the adduct is initiated by an incision (DSB formation) in the region surrounding the adduct. Unhooking of the ICL-adduct by XPF-ERCC1 proteins, DNA synthesis (directed by translesion DNA polymerases), and re-establishment of the replication fork integrity by HR, then completes repair of the DNA lesion [15], [17].

Analysis of NER protein expression in TGCT-derived cell lines revealed that levels of XPA, ERCC1 and XPF DNA repair proteins are reduced with respect to somatic tumors cells, suggesting that impaired NER function might account for TGCT sensitivity to ICL-inducing agents [9]. However, overexpression of ERCC1 and XPF in TGCT cell lines elicits only a small increase in their ICL-repair proficiency and resistance to cisplatin [10]. Therefore, while NER proteins appear to have a protective role, their deficiency does not fully explain the unique sensitivity of TGCTs to cisplatin and suggests that additional defects in ICL repair might be of critical significance.

Homologous recombination (HR) is an attractive candidate based on its importance for ICL repair; HR is frequently dysregulated in a variety of tymor types and HR deficiency is involved in the susceptibility of cancer cells to poly(ADP-ribose) polymerase (PARP) inhibitors. The cytotoxicity of PARP inhibitors has been postulated to result from an increased number of double strand breaks in cells that can poorly repair them [18], [19] and/or from dysregulation of the non-homologous end joining (NHEJ) pathway [20].

In this report, we show that the cisplatin-sensitivity of ECs is due to their inability to repair ICL-induced damage. Moreover, we demonstrate that in addition to defective ERCC1/XPF-dependent repair mechanisms, the sensitivity of TGCTs to ICL-inducing agents relies on their reduced proficiency in HR. Extending these findings, we tested the sensitivity of five EC cell lines (with varying degrees of cisplatin-sensitivity) to the PARP inhibitor AZD2281, either alone or in combination with cisplatin. Our results indicate that all EC cell lines are sensitive to AZD2281 monotherapy at clinically relevant concentrations and that inhibition of PARP activity significantly enhances cisplatin cytotoxicity especially in cell lines relatively resistant to the drug. These findings suggest that PARP inhibitors are a potential novel candidates for targeted treatment of ECs and TGCTs otherwise resistant to standard therapies.

## Results

### Embryonal Carcinoma (EC) Cell Lines Exhibit Differential Sensitivities to Cisplatin

**Figure 1:**
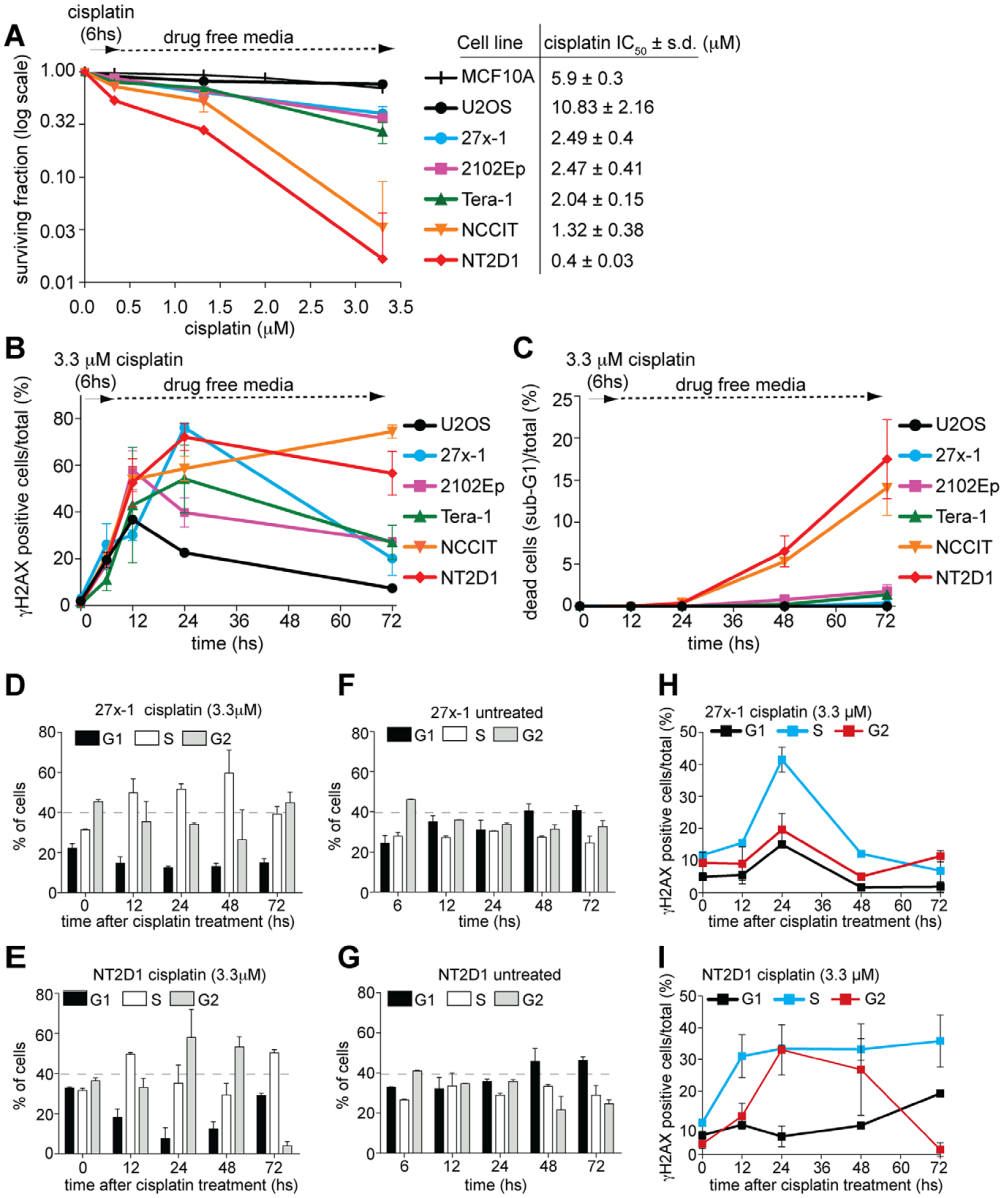
ECs are defective in ICL-induced damage repair. A) EC cell lines are sensitive to cisplatin treatment. Colony surviving assay of EC cell lines treated for 6 hs with the indicated concentrations of cisplatin. The surviving fraction was monitored by following colony formation for up to 14 days after treatment. Somatic cell lines MCF10A and U2OS were used as positive (resistant) controls. The table shows the IC_50_ value of cisplatin for each cell line. Data are mean value ± standard deviation (s.d.) of three to five independent experiments, each done in triplicate. B) The EC cell lines sensitivity to cisplatin correlates with their inability to repair ICL-induced DSBs**.** FACS profile of γH2AX-positive cells upon cisplatin treatment. EC cell lines were treated with 3.3 µM cisplatin for 6 hs and collected at the indicated time points, from the beginning of treatment, for γH2AX staining. Data are mean value ±s.d. of three independent experiments. C) The most cisplatin-sensitive EC cell lines are eliminated by apoptosis by 48 hs after treatment. The percentage of dead cells/total, following cisplatin treatment are shown. The indicated cell lines were treated as described in B and analyzed for their apoptotic elimination by FACS (sub-G1). Data are the mean value ± s.d. of three independent experiments. D–G) cell cycle profile of 27x-1 and NT2D1 cell lines following cisplatin treatment. The indicated cell lines were treated (or left untreated) as in B, and collected at the indicated time points for cell cycle analysis. In D–E t = 0 hs indicates the cell cycle profile of the cell lines at the end of cisplatin treatment (6 hs) and it is compared to t = 6 hs in F and G. Data are the mean value ± s.d. of three independent experiments H–I) distribution of γH2AX-positive cells trough cell cycle. Cells were treated as in B, collected at the indicated time points for the staining with the γH2AX antibody, and analyzed by FACS. Time t = 0 hs indicates the cell cycle profile of γH2AX-positive cells/total at the end of cisplatin treatment (6 hs). Data are the mean value ± s.d. of three independent experiments.

To determine the sensitivity of EC cell lines to cisplatin, we analyzed their ability to survive drug treatment by colony-forming assays (Fig. 1A). We compared five different cell lines, three of which, 27x-1 [21] NCCIT [22] and NTERA2-D1 (NT2D1) [23], are considered pluripotent, whereas the other two, 2102Ep and Tera-1 [24], [25], are classified as nullipotent (summarized in Table S1). As a control for cisplatin-resistant cells, we used the human osteosarcoma cell line U2OS and the non-tumorigenic epithelial cell line MCF10A. EC cell lines were sensitive to cisplatin compared with MCF10A and U2OS but displayed a wide range of response. The IC_50_ varied from 2.49±0.4 µM for the less sensitive cell line (27x-1) to 0.4±0.03 µM for the most sensitive cell line (NT2D1) (Fig. 1A). These results indicate that, although they appear more sensitive to cisplatin than somatic tumor (and non-tumor) cells, EC cell lines differ from each other, suggesting that resistant cells may have acquired specific molecular characteristics that render them less responsive to treatment.

### EC Cell Lines are Deficient in ICL-damage Repair

The phosphorylation of histone H2AX on serine 139 (γH2AX) is considered a general marker of DNA double strand breaks (DSBs) induced by several chemotherapeutic drugs, including cisplatin [26]. To analyze whether cisplatin-sensitivity of EC cell lines relies on reduced ability to repair DNA damage, we used Flow cytometry (FACS) analysis to monitor over time, the percent of cells that are γH2AX-positive, following a pulse-exposure to cisplatin (Fig. 1B). Unsynchronized U2OS and EC cells were treated for 6 hours (hs) with 3.3 µM cisplatin (a median plasma concentration measured in TGCT cisplatin-treated patients [27]) and collected at 12 h, 24 h and 72 hs after the start of cisplatin exposure. Cisplatin caused a cell cycle delay in S-phase in all cell lines, followed (in EC cells) by increased accumulation of cells in S or G2/M at the expense of cells in G1 (Fig. 1 D–G, and Fig. S1 A–H). In most cases, the initial cell cycle delay was accompanied by an increase of γH2AX signal in S-phase (Fig. 1 H–I, Fig. S1 I–L, Fig. S2); likely due to formation of DSBs at the collapsed replication-fork [15], [16]. However, while in the somatic tumor cell line U2OS γH2AX signal quickly decreased and returned nearly to control level by 72 hs, even in relatively cisplatin resistant EC cell lines (27x-1, 2102Ep, Tera-1), γH2AX reduction was much less efficient (Fig. 1B and H, and Fig. S1 I–K). Importantly, the different response of 27x-1, 2102Ep, Tera-1 and U2OS to damage was not attributable to differences in cell death, as the sub-G1 population was not markedly increased (Fig. 1C), indicating that these cell lines truly vary in their proficiency to repair ICLs. Furthermore, the most cisplatin-sensitive cell lines NCCIT and NT2D1 were even more defective in ICL repair such that the γH2AX signal increased over time after treatment (Fig. 1B and I and Fig. S1L), and a significant fraction of sub-G1 population arose (Fig. 1C). These results indicate that in EC cell lines, cisplatin sensitivity correlates with cellular proficiency to repair the ICL damage. Importantly, in all cell lines, the initial cisplatin-induced DNA damage (Fig. 1B, t = 6 h) was similar, suggesting that under our experimental conditions, cell lines are not significantly different from each other in drug uptake, efflux or detoxification [28].

### Reduced Proficiency of ECs to Repair ICL-induced Damage is Mechanistically Distinct from their Apoptotic Elimination

**Figure 2:**
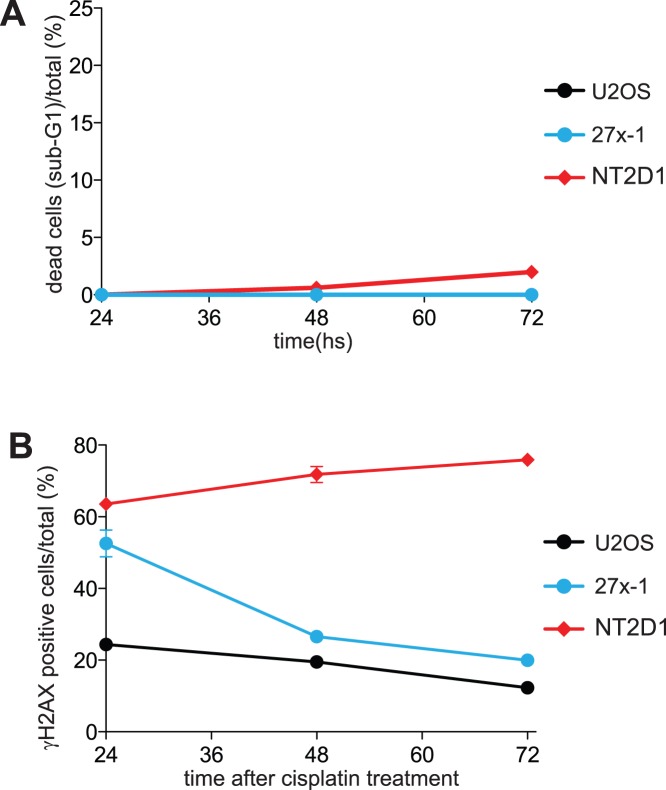
The inability of NT2D1 cell line to repair ICL-induced DSBs, is not rescued by preventing their apoptotic response. A) z-VAD-FMK treatment prevents NT2D1 cell line apoptotic response. The indicated cell lines were pretreated for 2 hs with z-VAD-FMK (50 µM) and then treated with cisplatin (3.3 µM) for 6 hs. At the end of incubation, cisplatin was removed, and the cells were maintained in constant presence of z-VAD-FMK for up to 24 hs, 48 hs and 72 hs after damage. At the end of treatment cells were analyzed for their apoptotic elimination (sub-G1) by FACS analysis. In the graph the apoptotic response of U2OS overlaps with that of 27x-1. Data are the mean value ± s.d. of three independent experiments. B) Flow cytometry of γH2AX-positive cells upon z-VAD-FMK and cisplatin co-treatment. Cells were treated as described in A, collected at the indicated time points, for γH2AX staining, and analyzed by FACS. Data are the mean value ± s.d. of three independent experiments.

It has been recently proposed that the inability of the NT2D1 cell line to repair cisplatin-induced damage is due to rapid and massive induction of apoptosis that precedes the onset of DNA damage-repair [8]. To evaluate whether in our experimental conditions apoptosis prevents damage repair and test whether increased γH2AX staining is due to DSB formation, rather than an the early apoptotic response, we analyzed cisplatin-induced γH2AX profile in the presence of the caspase-inhibitor, N-Benzyloxycarbonyl-Val-Ala-Asp(O-Me)-fluoromethyl-ketone (z-VAD-FMK). Unsynchronized cells were treated for 2 h with 50 µM caspase inhibitor followed by a pulse of 3.3 µM cisplatin for 6 h. After incubation, cisplatin was removed, and cells were cultured in the presence of z-VAD-FMK for up to 72 h after the start of cisplatin exposure. Under these conditions, cell death of NT2D1 was markedly reduced (compare Fig. 1C and Fig. 2A). However, the γH2AX signal did not decrease over time (Fig. 2B), indicating that EC cells have an intrinsic inability to repair ICL damage, which is mechanistically distinct from their apoptotic response. Notably, under the conditions described above, the cell cycle profiles were similar in all cell lines (data not shown). Thus the different kinetic of repair is not linked to different timing of cell cycle arrest.

### Reduced Expression of NER-repair Factors does not Fully Account for the Differential Sensitivity of EC Cells to Cisplatin

**Figure 3:**
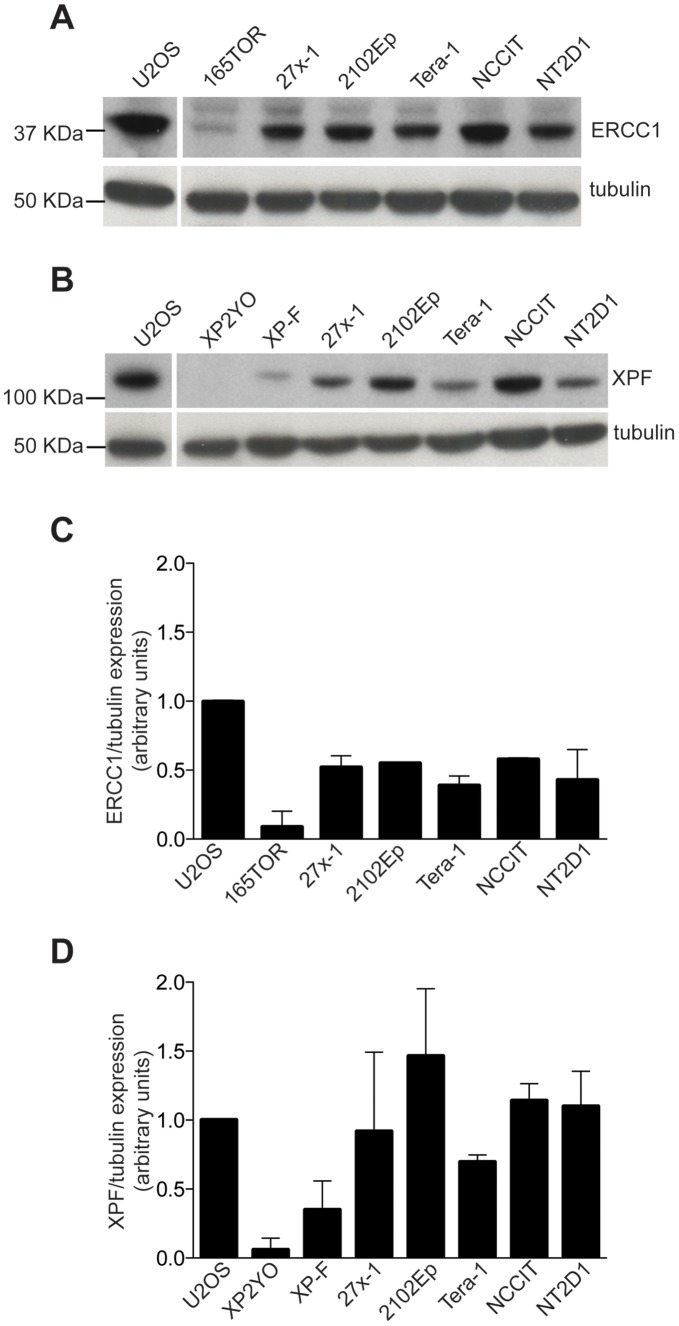
The high sensitivity of EC cell lines to cisplatin correlates with a reduced expression of ERCC1 but not XPF. A–B) Western blotting analysis of ERCC1 and XPF expression levels in five EC cell lines. U2OS, ERCC1-deficient 165TOR, XPF-deficient (XP2YO) and XP2YO-complemented (XP-F) cell lines were used as protein expression controls. β-tubulin was used as loading control. The U2OS lane comes from the same electrophoresis gel of the other cell lines. C–D) Densitometric analysis of ERCC1 and XPF expression. Results are presented as expression level respect to U2OS cell line, and normalized against the loading control (β-tubulin). Data are mean value ±s.d. of two independent experiments.

The reduced expression of NER-proteins XPF and ERCC1 has been proposed to explain TGCT sensitivity to DNA damaging agents [9], [10]. To examine this hypothesis, we analyzed our panel of EC cells for expression of XPF/ERCC1 proteins. As controls, we used three human fibroblast cell lines, one with significantly reduced expression of ERCC1 (165TOR) [29], one lacking XPF (XP2YO) [30] and a XP2YO cell line complemented with XPF (XP-F) [31]. Similar to a previous report [9], TGCT cell lines had somewhat reduced ERCC1 expression compared to U2OS (Fig. 3A and C); however, XPF protein levels were not obviously reduced in most cell lines (Fig. 3B and D). Because XPF and ERCC1 act as heterodimer, reduced expression in ERCC1 might drive the reduction in ICL-repair proficiency, promoting sensitivity of ECs to cisplatin. However, within EC cell lines, we did not observe a correlation between cisplatin resistance and ERCC1 expression levels. Since resolution of ICL damage requires the coordinated action of multiple repair mechanisms [17], this result suggests that the differential cisplatin-sensitivity of EC cells may depend on dysregulation of DNA repair mechanisms other than XPF-ERCC1.

### EC Cell Lines Display a Reduced Proficiency in HR

**Figure 4:**
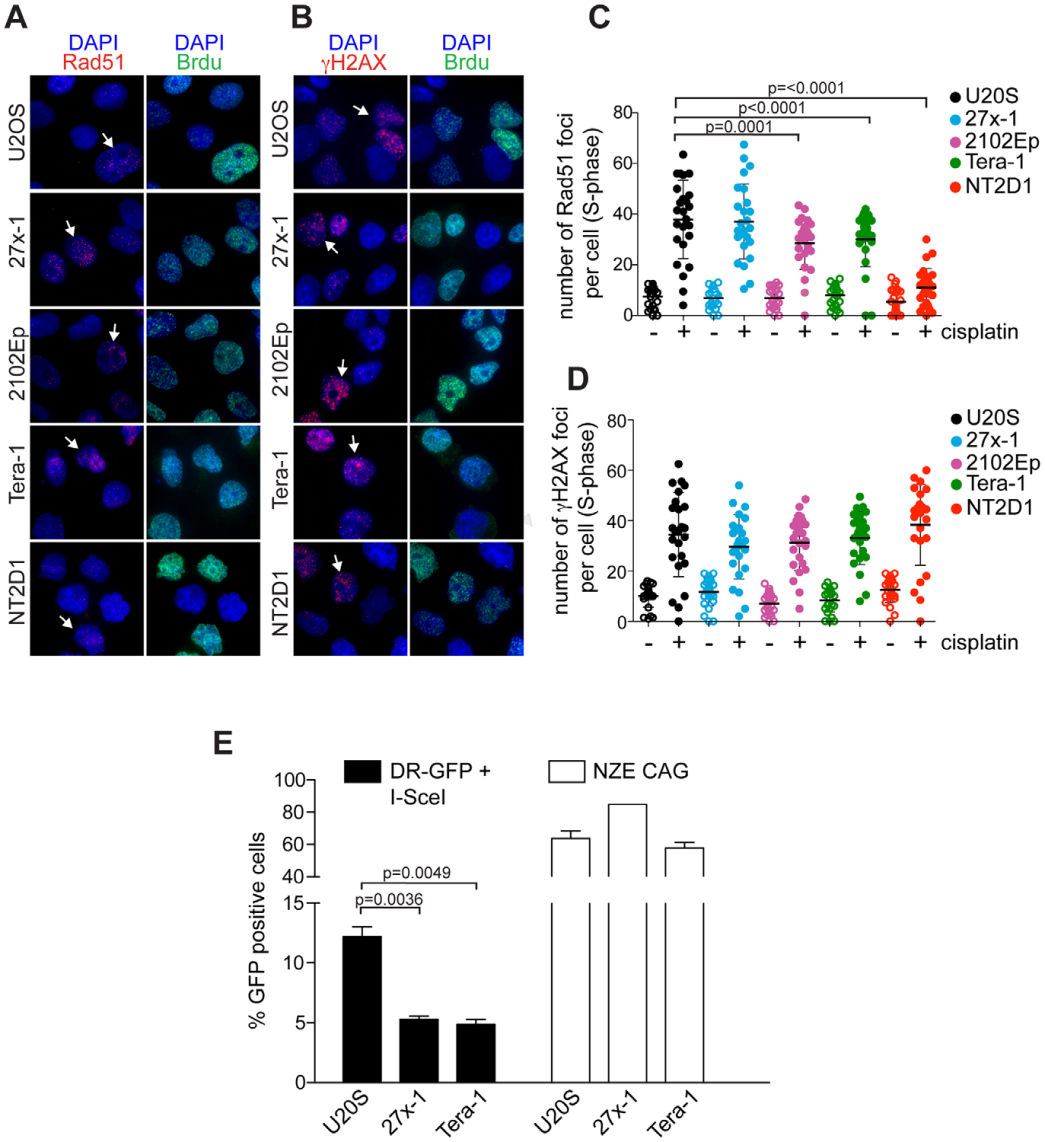
ECs are defective in HR repair. A) EC cells are defective in RAD51 foci assembly. The indicated cell lines were treated with a pulse of cisplatin (3.3 µM for 6 hs) and co-stained with anti-RAD51 (red) and anti-bromodeoxyuridine (BrdU, [green]) antibodies. Harrows points to representative BrdU-positive (S-phase) cells used for RAD51 quantification (see below). B) cisplatin induces a comparable damage in U2OS and EC cell lines. The indicated cell lines were treated as in A, co-stained with γH2AX (red) and BrdU (green) antibodies, and counterstained with DAPI (blue). Harrows points to representative BrdU-positive (S-phase) cells used for γH2AX quantification. C–D) quantification of the number of RAD51 (C) and γH2AX (D) foci, before and after cisplatin treatment. Data are mean value ±s.d. of two independent experiments. In C and D a minimum of 100 nuclei were counted for each cell line. Statistical analysis was performed using a paired two-tailed Student's *t*-test (P<0.05). E) 27x-1 and Tera-1 cell lines are defective in I-SceI-induced DSB,repair, by HR. Percentage of GFP+ cells measured by flow cytometry, 48 hs upon the transfection with both DR-GFP and I-SceI expression vectors (black bars, [DR-GFP+I-SceI]). Data were normalized against the transfection efficiency measured by transfecting a (constitutive) GFP-expressing vector (white bars [NZE CAG]). Data are mean value ± s.d. of three independent experiments. Statistical analysis was performed using a paired two-tail Student’s t-test (P<0.05). For more details see also Fig. S3A–B.

HR is a major pathway for repair of DNA ICLs in mammalian cells [15]. It functions in S/G2 phases in the same process with XPF/ERCC1, even though the latter are not required to initiate HR [17], [32]. At the cellular level, early steps of HR repair can be visualized (in both replicating and damaged cells) by the formation of RAD51 foci [33]. Since HR repair occurs during genome replication, we tested whether the sensitivity of EC cell lines to cisplatin was due to defective HR-repair by quantifying the number of RAD51 foci in S-phase. Cell lines were treated with 3.3 µM cisplatin for 6 h and co-stained with both anti-RAD51 and anti-bromodeoxyuridine (BrdU) antibodies. As shown in Fig. 4A and quantified in Fig. 4C, the number of RAD51 foci per cell was significantly lower in 2102Ep, Tera-1 and NT2D1 than in U2OS, indicating a defect in HR. In addition, sensitivity to cisplatin correlated with the level of S phase RAD51 foci; such that the most cisplatin-sensitive cell line, NT2D1, was the most defective in RAD51 foci assembly. Importantly, the observed reduction of RAD51 foci was not attributable to a difference in cisplatin-induced damage as documented by the similar number of γH2AX foci formed across cell lines (Fig. 4B, 4D and Fig. 1B [t = 6 hs]). The only exception was 27x-1, in which RAD51 foci numbers were not reduced with respect to U2OS. To test whether the HR-repair proficiency of 27x-1 was comparable to that of U2OS, we performed a functional HR-assay, measuring their ability to repair DSBs introduced by I-SceI in a GFP-recombination substrate (DR-GFP)[34]. In this assay, repair of DSB by HR results in reconstitution of a functional GFP gene such that HR-proficiency can be quantified by FACS analysis [35] (Fig.S3A). As a control, the assay was also performed in a cell line (Tera-1) in which cisplatin-induced RAD51 focus assembly was low. Consistent with their relative sensitivities to cisplatin, both 27x-1 and Tera-1 were two-fold less proficient in this assay than U2OS cells (Fig. 4E, black bars, and S3B). Transfection efficiencies are similar for all three cell lines (Fig. 4E, white bars). These data demonstrate that EC cell lines have a reduced proficiency in HR.

**Figure 5:**
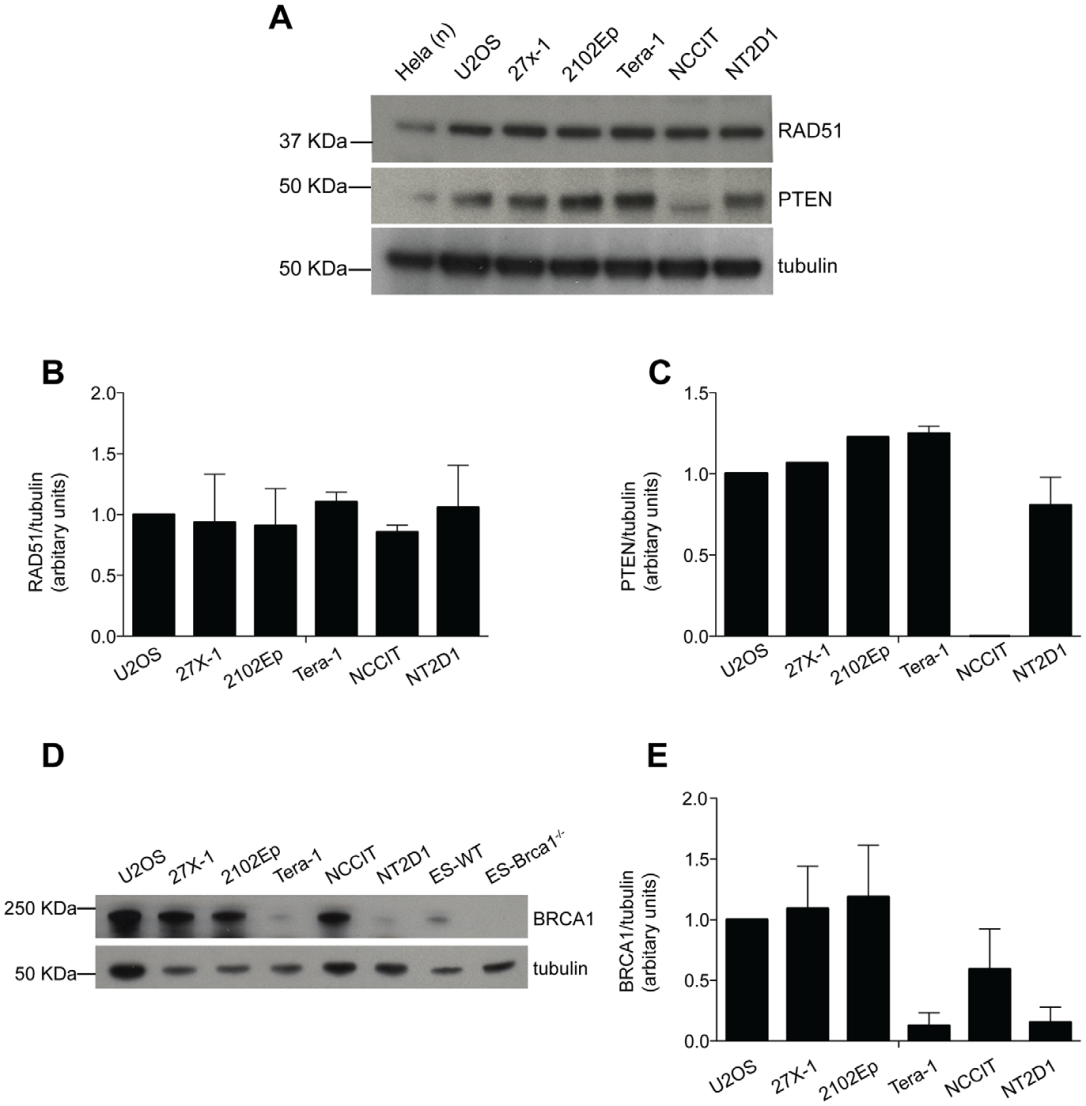
The reduced expression of RAD51, PTEN or BRCA1, does not account for the reduced proficiency of EC cell lines, in HR-repair. A) Representative images of western blotting analysis of RAD51 and PTEN in the indicated cell lines. The nuclear extract (n) of the somatic Hela cell line were used as positive control. β-tubulin was used as loading control. B) densitometric analysis of RAD51 expression in the indicated cell lines. Data are mean value ± s.d. of four independent experiments C) densitometric analysis of PTEN expression in the indicated cell lines. Note that the observed reduction of PTEN expression in NT2D1 cell line was not statistically significant when compared with any other EC cell line (unpaired t-test p<0.05). Data are mean value ± s.d. of two independent experiments. D) Representative images of a western blotting analysis of BRCA1 protein level in the indicated cell lines. Mouse embryonic stem cells (ES) wild type (WT) or knockout for Brca1 (*Brca1^−/−^*) were used as positive and negative controls respectively. E) densitometric analysis of BRCA1 expression of the indicated cell lines. Data are mean value ±s.d. of four independent experiments. In B, C and E, results are presented as expression level respect to U2OS cell line, and normalized against the loading control (tubulin).

To understand whether the defect in the assembly of RAD51 foci observed in most EC cell lines was the consequence of reduced expression of the protein, we performed a western blot analysis. RAD51 was similarly expressed in U2OS as in EC cell lines (Fig. 5A top panel, and Fig. 5B), indicating that a *functional* deficiency in formation of RAD51 foci rather than differential expression of this protein may cause an the HR defect. It has recently been suggested that deficiency in *phosphatase and tensin homolog* (*PTEN*) gene causes a functional defect in RAD51 foci assembly [36]. Because PTEN expression was reported to be virtually absent in 86% of ECs [37], we analyzed protein expression in our EC cell lines. As shown in Fig. 5A (mid panel) and quantified in Fig. 5C, PTEN was expressed in all cell lines except NCCIT, indicating that, as also suggested in another tumor model [38], reduced PTEN expression was not the primary cause of the defective RAD51 function.

The breast cancer tumor susceptibility gene product *BRCA1* has a fundamental role in HR [39], promoting proper RAD51 focus formation, including HR in ICL repair [14] [40]. Therefore, we analyzed BRCA1 protein expression in EC cell lines as compared to HR-proficient U2OS. As shown in Fig. 5D–E, BRCA1 expression was reduced, with respect to U2OS, in Tera-1 and NT2D1 cell lines, but not in 27x-1 and 2102Ep, and not significantly in NCCIT. Thus, although BRCA1 down-regulation might contribute to the increased cisplatin-sensitivity of Tera-1 and NT2D1, it does not appear to fully explain the differential response to cisplatin among EC cell lines.

### ECs are Sensitive to Treatment with the Poly (ADP-ribose) Polymerase Inhibitor AZD2281

**Figure 6:**
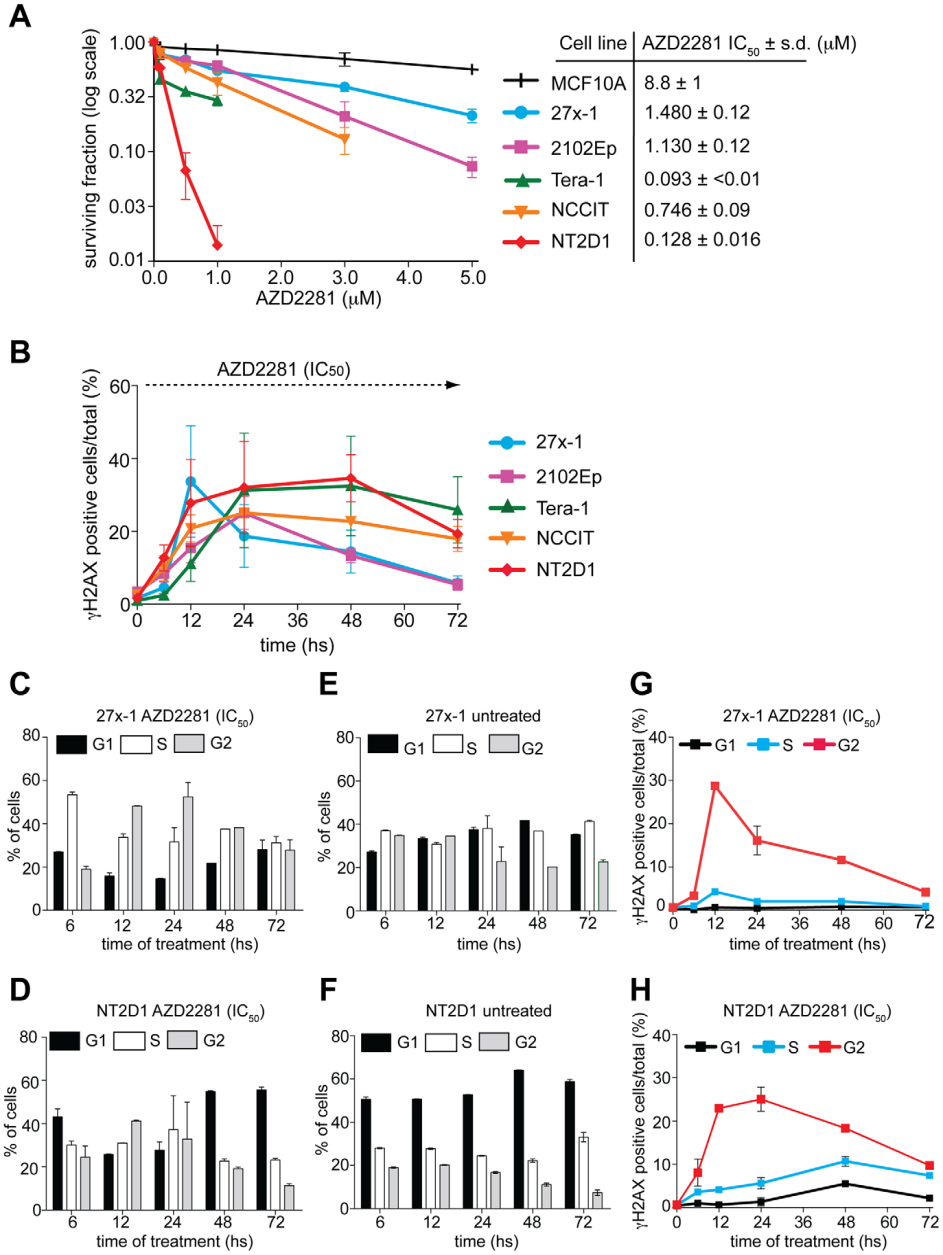
EC cell lines are sensitive to AZD2281 treatment. A) Colony assay of EC cells exposed over time to increasing doses of AZD2281. The somatic cell lines MCF10A was used as positive (resistant) control. The table shows the IC_50_ value of AZD2281 for each cell line. Data are mean value ± s.d. of three (triplicates) independent experiments. B) AZD2281 treatment induces DNA damage. FACS analysis of γH2AX-positive cells upon AZD2281 treatment. The indicated cell lines were treated over time with the IC_50_ dosage of AZD2281 and collected 6 hs up to 72 hs after initial treatment. Data are the mean value ± s.d. of two (triplicates) independent experiments. C–F) cell cycle profile of 27x-1 and NT2D1 cell lines following the treatment with AZD2281. The indicated cell lines were treated (or left untreated) as in B, and collected ad the indicated time points for cell cycle analysis. Data are the mean value ± s.d. of three independent experiments. G–H) cell cycle distribution of γH2AX-positive cells following AZD2281 treatment. Cells were treated as in B and collected, at the indicated time points, for the staining with the γH2AX antibody. Data are the mean value ± s.d. of three independent experiments.

Preclinical studies have demonstrated that cancer cells with defective HR repair caused by either *BRCA1* or *BRCA2* inactivating mutations, display exquisite sensitivity to PARP inhibitors [41], [42]. AZD2281 (olaparib; KU-0059436) is an orally active PARP inhibitor, which proved to be active and well-tolerated in preclinical mouse models [43] and in clinical trials with patients [44]. Since HR-reduced proficiency appears to be a common feature among EC cell lines, we analyzed their response to AZD2281. Recent evidence indicates that the treatment of U2OS with the PARP inhibitor PHEN down-regulates BRCA1/RAD51 expression causing a defect in HR [45]. By colony assay, we found that U2OS are also sensitive to AZD2281 (not shown), thus this cell line could not be used as resistant control cell line for this drug. To overcome this problem we compared the sensitivity of EC cell lines to AZD2281 to that of the relatively PARP inhibitor-resistant (and HR proficient) MCF10A [46], [47]. As shown in Fig. 6A, ECs were sensitive to AZD2281 treatment, and PARP inhibitor sensitivity generally correlated with sensitivity to cisplatin (compare Figures 1A and 6A). An exception was Tera-1, which was moderately resistant to cisplatin but extremely sensitive to AZD2281, suggesting that the mechanism(s) of resistance/sensitivity to platinum agents and PARP inhibitors do not completely overlap. Notably, however, the two most PARP inhibitor sensitive cell lines have very low BRCA1 levels (Fig. 5D).

To evaluate whether sensitivity of EC cell lines to AZD2281 correlates with their reduced proficiency to repair drug induced-DNA damage, we quantified the percentage of γH2AX positive cells, upon continuous exposure to the IC_50_ dose of AZD2281. As shown in Fig. 6B, while relatively PARP inhibitor-resistant cell lines (27x-1 and 2102Ep) were able to repair the damage, the most sensitive cell lines (Tera-1, NCCIT and NT2D1) displayed no reduction in γH2AX positive cells. As judged by the fraction sub-G1 cells, apoptosis was evident by 72 hs with the Tera-1 and NT2D1 cell lines (3.58±1.5% and 6.78±2.3%, respectively).

To further investigate the mechanism of EC cell sensitivity to PARP inhibition, we analyzed γH2AX levels throughout the cell cycle. In most EC cell lines (but not in MCF10A), AZD2281 treatment, was accompanied by a prominent increase in damage in G2 (Fig. 6G–H and Fig. S4I–L), with delay in the S/G2 phases of the cell cycle (Fig. 6C–F, and S4 A–H), indicating that the sensitization effect required passage through replication, a cell cycle stage when HR is more effective.

### Sensitivity of ECs to AZD2281 Correlates with PARP1 Protein Expression and Activity

**Figure 7:**
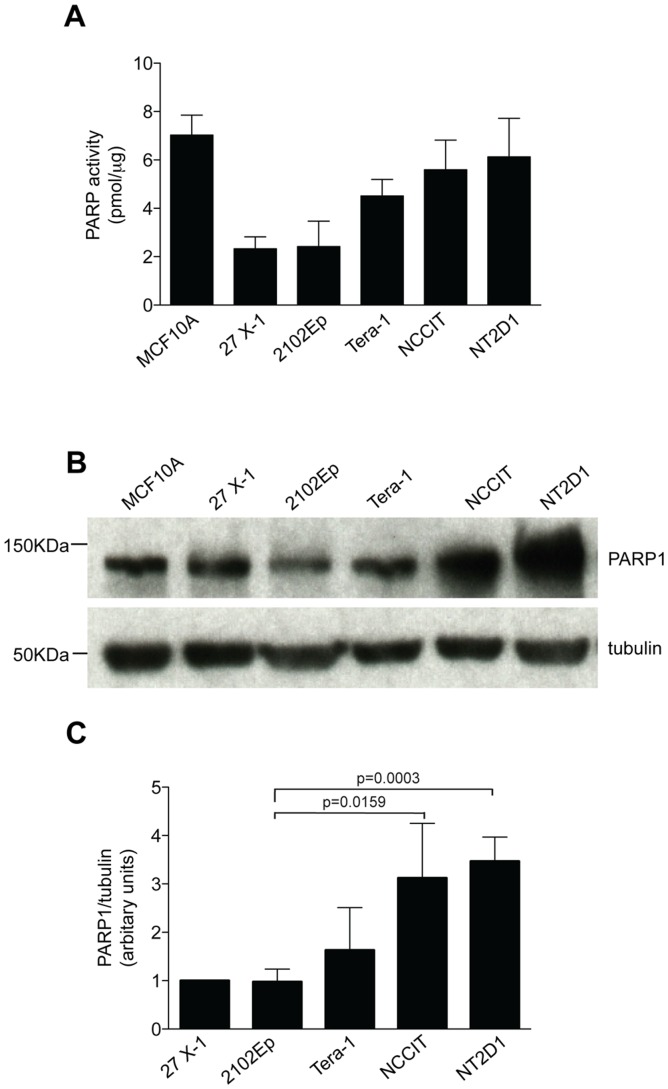
In EC cell lines PARP maximal activity correlates with PARP1 protein expression. A) PARP maximal activity correlates with EC sensitivity to AZD2281. The maximal PARP activity was assayed *in vitro* by measuring the levels of PAR (poly[ADP-ribose])-polymers linked to PARP (autoribosilatyon) and histone H1 (a PARP-1 substrate), in presence of activated DNA (nuclease treated DNA). Data are mean value ± s.d. of three to four independent experiments. B) Representative image of a western blotting analysis of PARP-1 expression, in the indicated cell lines. β-tubulin was used as loading control. C) densitometric analysis of PARP1 expression in the indicated cell lines. Data are the mean value ± s.d. of five independent experiments. Results are presented as expression level respect to 27x-1 cell line, and normalized against the loading control (β-tubulin). Statistical analysis was performed using a unpaired two-tail Student’s t-test (P<0.05).

PARP is frequently hyper-activated in HR-defective cells, and reversion mutations that rescue HR defects also reduce PARP activity [48]. Therefore, it has been proposed that DNA lesions that accumulate in HR-defective cells require PARP-mediated repair. There are at least 18 PARP family members described so far, however it is recognized that most cellular PARP activity (measured by PAR-polymer formation) is attributable to PARP1 [49]. We asked whether sensitivity of EC cell lines to AZD2281 correlates with PARP1 protein expression and activity. Using an *in vitro* assay, we measured the maximal PARP activity (induced by the presence of nuclease-treated DNA) and correlated the results with PARP1 protein expression levels. As shown in Fig. 7A, we found that within EC cell line group, PARP activity was higher in the most PARP inhibitor-sensitive cell lines (NCCIT, Tera-1, NT2D1). In addition, in most cell lines, PARP1 expression correlated with PARP inducible activity (Fig. 7B–C), suggesting that EC cell lines that rely more strongly on PARP activity to repair DNA damage (due to a reduced proficiency of HR) have increased PARP1 protein levels and are most likely to respond to PARP inhibitor monotherapy.

### AZD2281 Enhances the Sensitivity of TGCT Cell Lines to Cisplatin

**Figure 8:**
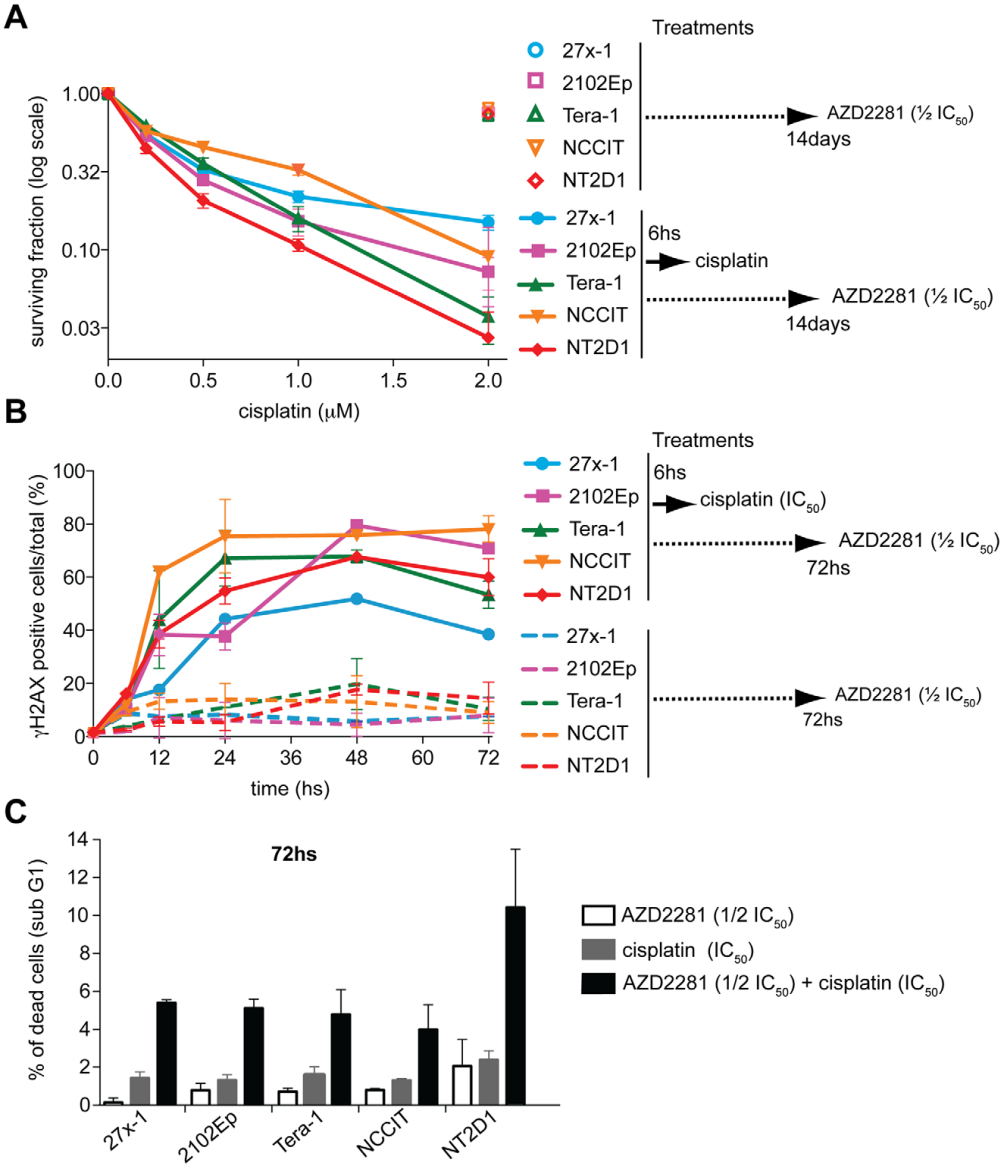
AZD2281 treatment enhances EC cell lines response to cisplatin. A) Colony assay. The indicated cell lines were treated with ½ of the IC_50_ dose of AZD2281 for 14 days, in absence (open symbols) or in presence (filled symbols) of cisplatin. In the latter case cisplatin was given for 6 hs, in presence of AZD2281 (½ of the IC_50_ dose). After initial treatment cisplatin was washed out and cells cultured for 14 days in presence of AZD2281. Data are mean value ±s.d. of three (triplicates) independent experiments. B) AZD2281 reduces the ability of EC cell lines to overcome cisplatin-induced damage. The indicated cell lines were either treated in continuous with the ½ of the IC_50_ dose of AZD2281 (dashed lines) or with AZD2281 plus the IC_50_ dose of cisplatin (non-dashed lines) as described in A) for up to 72 hs. At each indicated time point cells were harvested, and stained with the anti-γH2AX antibody for FACS analysis. Data are mean value ±s.d. of two independent experiments. C) AZD2281 enhances EC apoptotic response. The indicated cell lines were treated either with AZD2281 (½ of the IC_50_ dose, white bar) or cispaltin (IC_50_ dose, grey bar) or with combined therapy (black bar) as described in A, for up to 72 hs, and collected for FACS analysis of the sub-G1 fraction. Data are mean value ±s.d. of tree to four independent experiments.

Cisplatin and AZD2281 have been shown to cooperate in the treatment of BRCA-1 deficient mammary tumors *in vivo* [43], and PARP inhibitors enhance cancer cell sensitivity to radiation and alkylating agents [50]. Since we found ECs have a reduced proficiency in HR repair, we tested their sensitivity to combined therapy, exposing them to a pulse (6 hs) of increasing doses of cisplatin in continuous presence of AZD2281 (given at the half IC_50_ dose for each cell line). Under these conditions, the number of surviving colonies for the relatively cisplatin-resistant EC cell lines 27x-1 and 2102Ep was similar to that of cisplatin-sensitive NCCIT cells (Fig. 8A). The effect was even stronger for the Tera-1 cell line, whose survival profile paralleled that of NT2D1. This result indicates that AZD2281 enhance the toxicity of cisplatin in EC cells. Importantly the effect of the combined treatment was more marked in relatively cisplatin-resistant EC cells (27x-1, 2102Ep, Tera-1) than in the most cisplatin-sensitive (NCCIT, NT2D1) such that the IC_50_ value for cisplatin, was reduced of 2–3-fold in NCCIT and NT2D1, and about 10-fold in 27x-1, 2102Ep and Tera-1 (Table 1).

**Table 1:** Summary table indicating the IC_50_ value (µM) for AZD2281 (left column) and cisplatin, in absence (mid-column) or in presence (right column) of AZD2281.

Cell line	IC_50_ AZD2281	IC_50_ cisplatin(no AZD2281)	IC_50_ cisplatin(+1/2 IC_50_ AZD2281)
27x-1	1.48±0.12	2.49±0.4	0.26±0.05
2102Ep	1.13±0.12	2.47±0.41	0.23±0.01
Tera-1	0.093±<0.01	2.04±0.15	0.33±0.03
NCCIT	0.746±0.09	1.32±0.38	0.79±0.01
NT2D1	0.128±0.016	0.4±0.03	0.17±0.02

To gain further insight about the mechanism of EC-response to the combined treatment we analyzed the ability of cell lines to repair DNA damage using γH2AX as marker. The increased sensitivity of EC cell lines to combined treatment was linked to their inability to repair DNA (Fig. 8B), damage, and DNA-damaged cells arrested at G2 phase of the cell cycle (Fig. S5). As result, the percentage of apoptotic cell death as measured 72 hs after the beginning of treatment was increased with respect to either cisplatin or AZD2281 monotherapy treatments (Fig. 8C), validating the effect of the combined therapy.

## Discussion

Embryonal carcinomas (ECs) are considered the stem cell component of nonseminomatous TGCTs and, as cancer stem cells, maintain self-renewal capacity and are often multipotent. Along with vascular invasion, the concomitant presence of over 50% EC cell type in the primary tumor, is a risk factor for tumor relapse in patients with stage 1 nonseminomatous TGCT [5], [6]; probably because in most cases the invading element is the EC [7]. For these patients, the development of post-operatory (orchiectomy) adjuvant therapy to delay or prevent tumor relapse is needed. In addition despite the high curability rate, in some cases TGCTs become resistance to treatment. Thus, the development of new therapies to overcome resistance is also urgently required.

We have studied five EC cell lines, and characterized their sensitivity to cisplatin, demonstrating that these cells are more sensitive to cisplatin compared to the somatic cell lines MCF10A and U2OS. Nevertheless, within EC cell line the sensitivity to cisplatin also varied, with 27x-1, 2102Ep and Tera-1 being more resistant than NCCIT and NT2D1 (Fig. 1A). This observation suggests that ECs can have or acquire molecular characteristic that render them more resistant to treatment, increasing the risk of incomplete response and tumor relapse.

Why are EC cells generally more sensitive to DNA damaging agents than somatic tumors and why are some EC cell lines more sensitive to cisplatin than others? Chemosensitivity can be influenced by several factors, including drug transport across the cell membrane, drug detoxification, and accessibility of drug to DNA. We observed that the initial cisplatin-induced DNA damage measured by γH2AX staining (Fig. 1B [t = 6 hs] and Fig. 4D), is not significantly different in EC cell lines compared to the somatic tumor cell line U2OS; indicating that the superior responsiveness of EC cells to cisplatin is not attributable to upstream events that regulate cisplatin damage to DNA.

*TRP53* is intimately involved in the induction of apoptosis, cell cycle arrest and resistance to therapy following DNA damage. This gene is often mutated in somatic tumors but only rarely in TGCTs [51]. This difference has been proposed as one explanation for the unique cisplatin sensitivity of TGCTs. However, the high sensitivity of *TRP53*-mutated NCCIT cells (Table S1) to cisplatin observed here (Fig. 1A) and in other studies indicates that *TRP53* mutation alone does not confer cisplatin-resistance in these cells [51]. In addition, as previously reported in other studies [8], neither high constitutive p53 protein levels nor p53 accumulation/activation upon cisplatin treatment correlated with EC cell lines sensitivity, (Fig. S6) suggesting that EC cells possess unknown intrinsic characteristics that make them unique in their response to DNA damage.

Recently, Gutekunst and colleagues proposed that the extreme platinum sensitivity of EC cell lines is due to p53 protein level increases, induced either by DNA damage (cisplatin) or by MDM2-inhibitors (Nutlin-3), resulting in a rapid and massive apoptotic response that prevents DNA repair [8]. However, in our study, prevention of the apoptotic response did not influence the ability of EC cells to repair DNA damage (Fig. 2A–B). These discrepant results could reflect the fact that the methods used by these authors likely preferentially detect intrastrand (which account for over 90% of cisplatin lesions) rather than ICLs. TGCTs have been reported to be proficient in intrastrand cross-link, but not ICL repair [10] [8], supporting this conclusion. In addition, similar to recent findings [52] [44], we found that, in EC cells, Nutlin-3 induces DNA-damage, as measured by γH2AX staining (data not shown). Thus, although we agree that increased levels of p53 cause activation of a rapid and massive apoptotic response in EC cells, we believe that the trigger for this response is the persistence of DNA damage in cells that can poorly repair it. In this context, while the development of TGCTs would be allowed by a partial functional inactivation of p53 (see [53], [54]), such mechanism would be insufficient to counteract the pro-apoptotic function of p53 induced by a persistent damage, causing a rapid cell death. The observation that co-treatment of cells relatively resistant to cisplatin with cisplatin and AZD2281 causes a defect in repair of ICLs (persistent damage) and reduces their ability to form colonies (Fig. 8), supports this conclusion.

Which defect in DNA damage repair is most critical to cisplatin sensitivity in EC cells? Usanova observed that the overexpression of ERCC1 and XPF could partially reverse the cisplatin-sensitivity of TGCT cell lines [10], suggesting that a reduced proficiency in ICL-repair, induced by the low expression of these factors, in EC cells might promote cisplatin sensitivity [9]. In the current study, we observed an approximately 50% reduction in ERCC1 (but not XPF) expression as compared to U2OS (Fig. 3). However, since U2OS can be up to 27-fold more resistant than ECs to cisplatin (Fig. 1A), this difference is unlikely to fully account for the platinum-sensitivity of EC cell lines, suggesting that additional mechanisms of ICL-repair might be defective. In addition, among EC cell lines, there was no correlation between levels of ERCC1 expression and cell sensitivity to DNA damage.

Because the ability of cycling cells to repair ICLs requires HR [15], [17], we hypothesized that EC cells may be defective in this pathway. Indeed, we observed that 2102Ep, Tera-1 and NT2D1 were defective in RAD51 foci assembly (Fig. 4A and 4C), indicating a defect in HR repair. The EC cell line 27x-1 which is equally sensitive to cisplatin as 2102Ep and Tera-1 (Fig. 1A) was not defective in this assay, but was equally defective as Tera-1 cells in repair of ISce-I induced DSBs (Fig. 4E and Fig. S3B). These results suggest that perhaps 27x-1 cells are defective in a step downstream of RAD51 foci formation, and more in general that ECs are functionally defective in HR-repair, as also suggested by the similar expression of RAD51 in all cell lines (Fig. 5A–B). However, overall the molecular mechanisms behind EC HR-reduced proficiency are still unclear and might be different among cell lines. Their identification will likely require high throughput studies to globally examine the gene expression signature that distinguishes TGCT cells from other solid tumor cells that display lower sensitivity to cisplatin treatment.

HR-deficient tumors can be specifically targeted by treatment with PARP inhibitors [18], [19]. Therefore, we investigated whether EC cell lines are responsive to PARP inhibition. As shown in Fig. 6A, EC cells were sensitive to AZD2281 and their response correlated with reduced ability to overcome PARP inhibitor-induced damage (Fig. 6B). Interestingly, in most cases, the response to treatment with AZD2281 correlated with the magnitude of HR defect; such that 27x-1 and 2102Ep, which were relatively proficient in this pathway, were more resistant as compared to grossly defective NT2D1 cells. Because AZD2281-treated cells experience a delay in S/G2 phases of the cell cycle (Fig. 6C–F and S4A–H) and the induced-damage predominantly occurs in G2-phase (Fig. 6G–H and Fig. S4I–L), it is likely that DSB formation requires passage through S-phase; possibly as a consequence of inhibition of the base excision repair pathway by the inhibition of PARP [41]. However, dysregulation of the NHEJ pathway might also play a role [20]. Interestingly, Tera-1 cells, which are relatively resistant to cisplatin, are extremely sensitive to PARP inhibition (Fig. 6A). One possible explanation is that in these cells PARP activity is required to sustain HR function, as previously demonstrated in U2OS and other cell lines [45]. In addition, the observation that PARP-inducible activity is higher in the most PARP inhibitor-sensitive EC cells (Fig. 7A) suggest that in these tumors PARP activation might be required to compensate for the HR defect. Interestingly, PARP1 protein expression correlates with its inducible activity in most EC cells, (Fig. 7B–C) and thus might represent a predictive marker for response to PARP-inhibitors monotherapy in EC cells despite its lack of predictive ability in other tumor models [55]. The observation that EC cells are sensitive to AZD2281 monotherapy might also be of interest in the clinical setting to delay or prevent tumor relapse. In TGCT patients, tumor recurrence varies depending on the risk-group. Vascular invasion and high percentage (>50%) of EC are known predictors of metastases in stage 1 TGCT patients, especially when the two risk factors are present concomitantly (high-risk group) [5], [6]. Because in the latter case, the EC component of the tumor is often the invading element [7], and high percentage/pure EC plus vascular invasion is a common feature, we propose that patients that present both risk factors might be potentially eligible, and take advantage from a (post-orchiectomy) adjuvant chemotherapy treatment, which includes AZD2281. Such treatment, might represent a valid option to reduce or prevent disease relapse.

Remarkably, we found that AZD2281 enhances cisplatin cytotoxicity, most dramatically in relatively platinum-resistant EC cell lines (Fig. 8A and Table 1), even at concentrations of AZD2281 below the plasma concentration measured in clinical trials [42]. Such response is due to the reduced ability of the cells to repair overcome the damage (Fig. 8B) and increased apoptotic cell death (Fig. 8C), likely caused by the inability of the cells in S/G2 phase of the cell cycle to process ICL-induced DSB properly (Fig. S5).

In conclusion, the findings that EC cells have a reduced proficiency in HR and are sensitive to PARP inhibitor AZD2281 monotherapy suggest that this drug might be of interest for the treatment of patients with high-risk of occult metastasis. In addition, the observation that AZD2281 enhances cisplatin sensitivity in relatively-resistant EC cells, suggests that PARP inhibitors might be used to implement TGCT therapy, especially in patients resistant to standard therapies.

## Materials and Methods

### Cell Lines and Culturing

U2OS and TGCT cell lines were cultured in DMEM 15% FCS plus antibiotics (Lonza). EC cell lines were provided by R. S. K. Chaganti (Memorial Sloan-Kettering Cancer Center) [21], [22], [23], [24], [25]. Cell line 2102Ep was also provided by Prof. P. Andrews (Sheffield University, GB). U2OS was obtained from Amerycan Type Culture Collection (ATCC). The primary human fibroblast XP2YO and XPF-complemented (XP-F) cell lines were provided by Dr. L.J. Niedernhofer (Pittsburgh University); while cell line 165TOR was provided by Dr. Jan Hoeijmakers (Erasmus MC). Cell lines were cultured as described [29], [30], [31], [56]. Wild type and *Brca1^−/−^* Mouse embryonic stem cells (ES) were cultured as described in [35]. All cell lines were maintained at 37°C in humidified atmosphere with 5% CO_2_.

### Clonogenic Cell Survival Assay

2×10^3^ to 3×10^3^ cells were seeded in 10 cm plates, and allowed to adhere at for 18 hs before drug treatments. Cisplatin (Sigma Aldrich) was dissolved in dimethyl sulfoxide (DMSO) and given alone or in combination with AZD2281 for 6 hs. At the end of the incubation drugs were washed out, and cells cultured for 14 days in either drug-free media or in constant presence of AZD2281. AZD2281 (Organic Synthesis Core Facility, MSKCC) was dissolved in DMSO and added fresh into the culture every 2 days. At the end of treatment, cells were fixed in methanol, and stained with Giemsa 20% (Sigma Aldrich) for quantification of the number of colonies.

### Flow Cytometric (FACS) Quantification of Phospho-H2AX (Ser139)

Approximately 500×10^3^ cells either treated or untreated with cisplatin, and/or AZD2281, were collected, fixed in cold 70% ethanol, washed in Tris-buffered saline pH 7.4 (TBS) and rehydrated for 10 min at room temperature in TBS with 4% BSA and 0.1% Triton X-100 (TST). The primary antibody, anti-phosho-Histone H2AX (Ser139) (Upstate Biotechnology), was diluted 1∶250 in TST, and incubated for 2 hs at room temperature (RT). After two washes with TBS buffer, cells were incubated with the secondary antibody diluted 1∶200 (FITC-conjugated Invitrogen Alexa Four 488) in TST for 1 h at RT. Cells were then rinsed in TBS and resuspended in TBST/50 mg/ml RNase A in presence of 100 mg/ml propidium iodide (PI; Sigma-Aldrich) and incubated for 1 h at 37°C. For cell cycle analysis, a minimum of 10×10^3^ stained cells were acquired on a FACScan (Becton Dickinson) and analyzed with the Flowjo software.

### Immunoblotting

Upon trypsinization, cells were washed in PBS, and resuspended in 300 µl NETT lysis buffer (100 mM NaCl, 50 mM Tris base, pH 7.5, 5 mM EDTA, pH 8.0, 0.5% Triton X-100) containing Complete mini-protease and phosphatase inhibitor cocktails (Roche Molecular Biochemicals). Antibody sources and dilutions were as follows: RAD51 H-92 (1∶500), p53 sc-263 (1∶1000), PARP1/2 H-250 (1∶1000) and BRCA1 C-20 (1∶250), Santa Cruz Biotecnology. P21 CP-74 (1∶500), Thermo scientific. XPF ab-76948 (1∶1,000), Abcam. ERCC1 NB-100–117 (1∶1,000). Anti-tubulin DM1A T-9026 (Novus Biologicals) and anti-actin A-5316 (Sigma Aldrich) antibodies were used at 1∶15,000 dilution. Densitometric analysis of immunoblottings were performed by using the ImageQuant 5.1 software (Amersham).

### RAD51 and γH2AX Foci Assay

RAD51 and γH2AX foci were quantified in S-phase staining for BrdU. To do so, cisplatin-untreated or treated cells were exposed to 10 µM BrdU (Sigma Aldrich) 30 minutes before fixation with 4% paraformaldehyde. After fixation, cells were washed in PBS and incubated with 1 M HCl for 20 minutes. Following a wash with PBS, cells were incubated in 0.1 M sodium borate (PH 8.5) for 2 minutes at RT and washed again with PBS. Permeabilization was performed with PBS 0.5% Triton X-100 plus 0.5% normal goat serum (NGS) at RT for 15 minutes, and followed by blocking with 0.2% NGS in PBS at RT for 1h. The anti-RAD51 (Santa Cruz) γH2AX (Cell Signaling) and anti-BrdU (33281A, Pharmigen) antibodies were used at 1∶250, 1∶1000, and 1∶200 dilutions respectively. Primary antibodies were incubated for 90 minutes at RT. Secondary antibodies (Alexa-488 and Alexa-594, Invitrogen) were used at 1∶1000 dilutions. The total number of foci in BrdU positive and negative cells, were counted manually by using Zeiss observer Z1 microscope. All images were acquired at 100x magnification.

### DSB Assays

DSB repair was measured by using a GFP-based assay for HR as described previously [35] In brief, efficiency of HR was assessed by co-transfecting an I-SceI expression plasmid (pCBASce) with a GFP-reporter substrate (DR-GFP). The assay works through gene conversion repair of a DSB caused by I-SceI digestion; such that the DR-GFP plasmids repaired by HR express GFP (see Fig. S3A). U2OS and EC cells were transiently transfected (Amaxa Biotechnology) with 1 µg of DR-GFP plus 3 µg of I-SceI expressing vector or 1 µg of DR-GFP plus 3 µg of control plasmids (pCAGGS). Transfection efficiency was evaluated by transfecting cells with 1 µg of a GFP-expressing vector (Nze-GFP) plus 3 µg of pCAGGS. The number of GFP-expressing cells and cell cycle profiles were evaluated using the Becton Dickinson FACScan, and analyzed with the Flowjo software.

### PARP Activity Assay

Cells were lysed in 50 mM Tris-HCl buffer, pH 8, containing 0.6 mM EDTA, 14 mM β-mercaptoethanol, 10 mM MgCl_2_, 0.1% Triton X-100 and cocktail of proteases inhibitors (Roche) as previously described [57]. Proteins (25 µg) were incubated with 2 mCi ^32^PNAD^+^, 100 mM NAD^+^, 100 mg/mL H1 histone, 50 mM Tris-HCl, 10 mM MgCl_2_, and 14 mM β-mercaptoethanol in the presence of 10 µg nuclease-treated salmon testes DNA (Enzo Life Science). After 15 minutes at 30°C the reaction was stopped by addition of ice-cold trichloroacetic acid 20% (v/v) and the radioactivity associated with the acid-insoluble material, corresponding to poly(ADP-ribosyl)ated proteins, was counted on a Beckman LS8100 liquid scintillation counter. PARP activity was expressed as pmol of ^32^P-NAD^+^/mg of protein.

## Supporting Information

Figure S1 **Cell cycle distribution and mean percentage of γH2AX positive cells in G1, S and G2 phases of the cell cycle in exponential phase populations of U2OS and EC cell lines treated (or untreated) with cisplatin.** A–D) Cell cycle profile following cisplatin-induced damage. Cells were treated with a pulse of 3.3 µM cisplatin for 6 hs, collected at the indicated time points after treatment, and stained with propidium iodide for FACS analysis. Time t = 0 hs indicates the cell cycle profile of cells at the end (6 hs) of cisplatin treatment. E–H) Cell cycle distribution in absence of cisplatin. I–L) cell cycle distribution of γH2AX-positive cells/total following cisplatin treatment. The indicated cell lines were treated as described above, collected at the indicated time points after treatment, and stained with the anti-γH2AX antibody for FACS analysis.
(TIF)

Figure S2 **DNA double strand breaks after cisplatin treatment occurs in S/G2 phases of the cell cycle.** Representative distribution of γH2AX staining in the indicated cell lines, treated (D–F), or left untreated (A–C) with cisplatin. Cisplatin was given as a pulse of 3.3 µM cisplatin for 6 hs. Cells were collected 24 hs after the beginning of treatment, and stained with propidium iodide (PI) [x axis] and γH2AX antibody (y axis), for FACS analysis. Please note that, in EC cells, γH2AX signal increases dramatically in S/G2 phase upon cisplatin treatment.
(TIF)

Figure S3 **DR-GFP assay**. A) Schematic representation of the DR-GFP substrate. The DR-GFP gene is a modified GFP gene in which GFP is modified to *SceGFP* (cassette 1) so as to contain an ISceI site (incorporated at the BcgI site) and in frame termination codons. Downstream of the *SceGFP* gene, is an internal GFP fragment (cassette 2). Repair of DR-GFP substrate by homology-direct repair (HR) restore GFP function. B) Representative flow cytometry profile of the indicated cell lines analyzed 48 hs following plasmids transfection. Neg =  GFP profile of cells transfected with DR-GFP plasmid plus a control plasmid (pCAGGS). I-SceI =  GFP profile of cells transfected with DR-GFP plasmid plus a I-SceI expression plasmid (pCBASce). The circled area indicates the GFP+ cells. NZE CAG =  GFP profile of cells transfected with a GFP expressing plasmid (Nze-GFP). The percentage of DR-GFP positive cells was normalized against the percentage of Nze-GFP positive cells (transfection efficiency).
(TIF)

Figure S4 **Cell cycle distribution and mean percentage of γH2AX positive cells in G1, S and G2 phases of the cell cycle in exponential phase populations of U2OS and EC cell lines treated (or untreated) with AZD2281**. A–D) Cell cycle distribution following AZD2281 treatment. Cells were treated in continuous with the IC_50_ dose of AZD2281, collected at the indicated time points, and stained with propidium iodide for FACS analysis. E–H) cell cycle distribution of the indicated cell lines in absence of drug treatment. I–L) Cell cycle distribution of γH2AX-positive cells following AZD2281 treatment. The indicated cell lines were treated as described above, collected at the indicated time points, and stained with the anti-γH2AX antibody for FACS analysis. Data are mean value ± s.d. of three independent experiments.
(TIF)

Figure S5 **Cell cycle distribution and mean percentage of γH2AX positive cells in G1, S and G2 phases of the cell cycle in exponential phase populations of U2OS and EC cell lines treated (or untreated) with cisplatin/AZD2281 combined therapy**. A–E) Cell cycle distribution following cisplatin/AZD2281-combined treatments. Cells were co-treated with cisplatin (at a concentration corresponding to the IC_50_ of each cell line) and AZD2281 (at a concentration corresponding to the ½ IC_50_ of each cell line) for 6 hs. At the end of treatment cisplatin was washed out and cells maintained in continuous presence of AZD2281 (½ IC_50_ dose). Cells were collected at the indicated time points, and stained with propidium iodide for FACS analysis. F–J) cell cycle distribution of the indicated EC cell lines in absence of drug treatment. K–O) Cell cycle distribution of γH2AX-positive cells following cisplatin/AZD2281 combined treatment. The indicated cell lines were treated as described above, collected at the indicated time points, and stained with the anti-γH2AX antibody for FACS analysis. Data are mean value ± s.d. of three independent experiments.
(TIF)

Figure S6 **The status of** ***P53*** **does not predict EC sensitivity to cisplatin.** Representative images of a western blotting analysis of p53 and p21 protein levels, following 24 hs treatment with 3.3 µM cisplatin. All cell lines but NCCIT (not shown) are wild-type for *TRP53*, as shown by the increase of both p53 and p21 proteins level upon cisplatin treatment. Actin was used as loading control.
(TIF)

Table S1 **Origin of EC cell lines used, with their** ***P53*** **status.** Cell lines are predicted to be either mutant (NCCIT) or wild type (2102Ep, Tera-1, NT2D1) for*TRP53*. 27x-1 cell line is also wild type for p53, as shown by p53 (and p21) up-regulation upon cisplatin treatment (see Fig. S6).
(TIF)
