# Supplementary figures and images for: Reduced Proficiency in Homologous Recombination Underlies the High Sensitivity of Embryonal Carcinoma Testicular Germ Cell Tumors to Cisplatin and Poly (ADP-Ribose) Polymerase Inhibition
Source: PLoS One. 2012 Dec 12;7(12):e51563. doi: 10.1371/journal.pone.0051563 (PMC3520950; doi:10.1371/journal.pone.0051563)

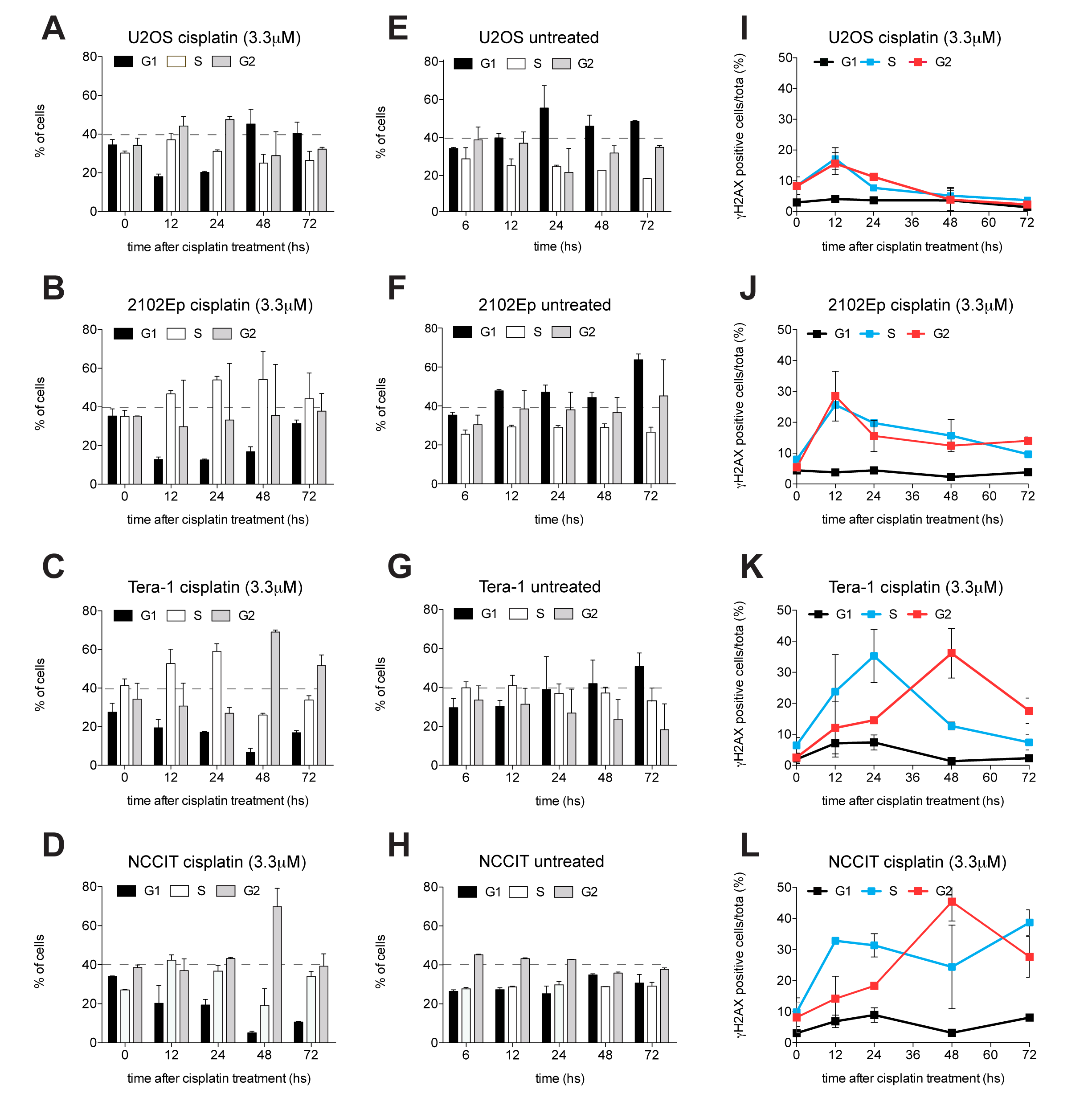

Supplement: Figure S1 — Cell cycle distribution and mean percentage of γH2AX positive cells in G1, S and G2 phases of the cell cycle in exponential phase populations of U2OS and EC cell lines treated (or untreated) with cisplatin. A–D) Cell cycle profile following cisplatin-induced damage. Cells were treated with a pulse of 3.3 µM cisplatin for 6 hs, collected at the indicated time points after treatment, and stained with propidium iodide for FACS analysis. Time t = 0 hs indicates the cell cycle profile of cells at the end (6 hs) of cisplatin treatment. E–H) Cell cycle distribution in absence of cisplatin. I–L) cell cycle distribution of γH2AX-positive cells/total following cisplatin treatment. The indicated cell lines were treated as described above, collected at the indicated time points after treatment, and stained with the anti-γH2AX antibody for FACS analysis. (TIF) [file pone.0051563.s001.tif]

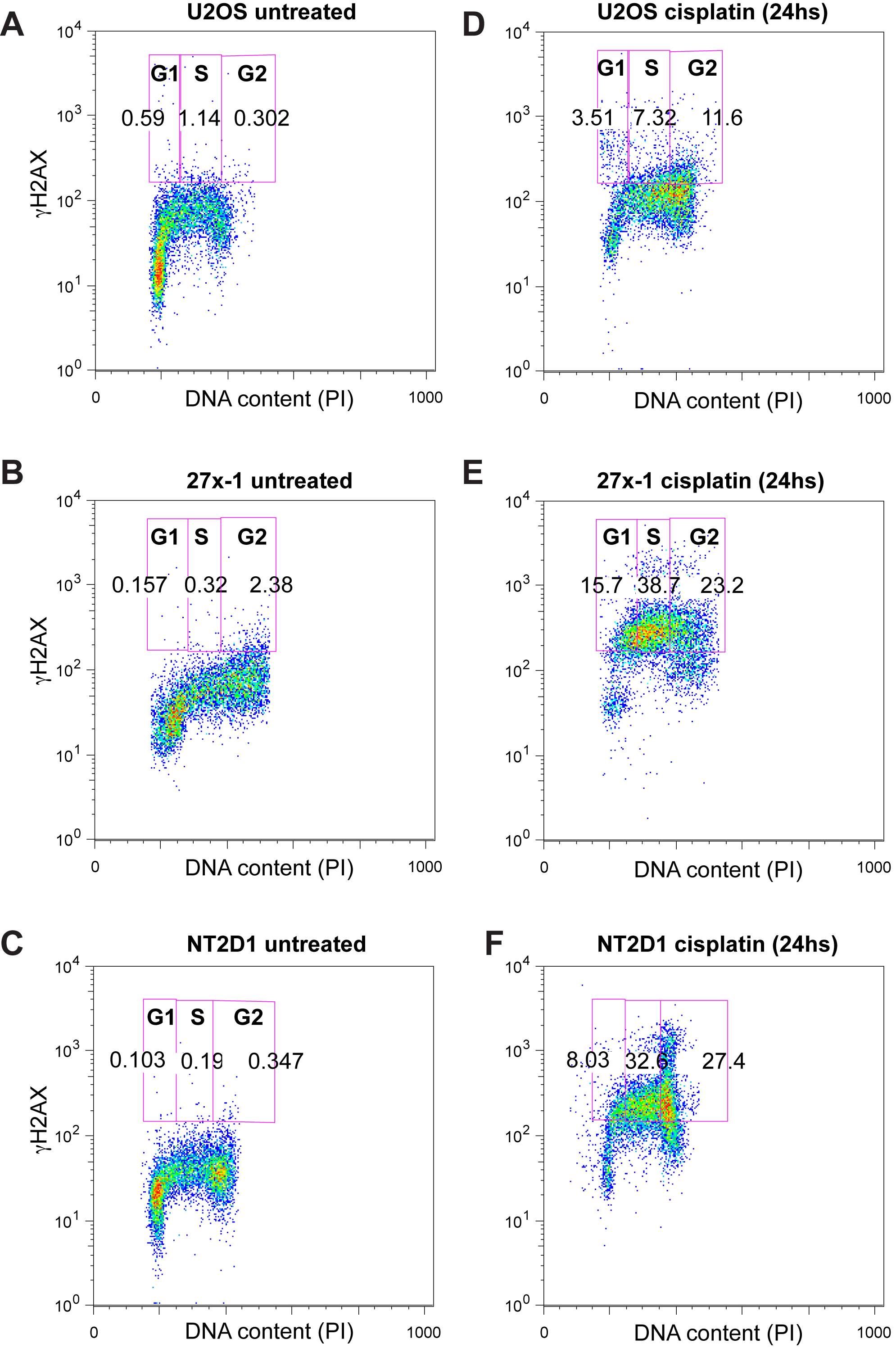

Supplement: Figure S2 — DNA double strand breaks after cisplatin treatment occurs in S/G2 phases of the cell cycle. Representative distribution of γH2AX staining in the indicated cell lines, treated (D–F), or left untreated (A–C) with cisplatin. Cisplatin was given as a pulse of 3.3 µM cisplatin for 6 hs. Cells were collected 24 hs after the beginning of treatment, and stained with propidium iodide (PI) [x axis] and γH2AX antibody (y axis), for FACS analysis. Please note that, in EC cells, γH2AX signal increases dramatically in S/G2 phase upon cisplatin treatment. (TIF) [file pone.0051563.s002.tif]

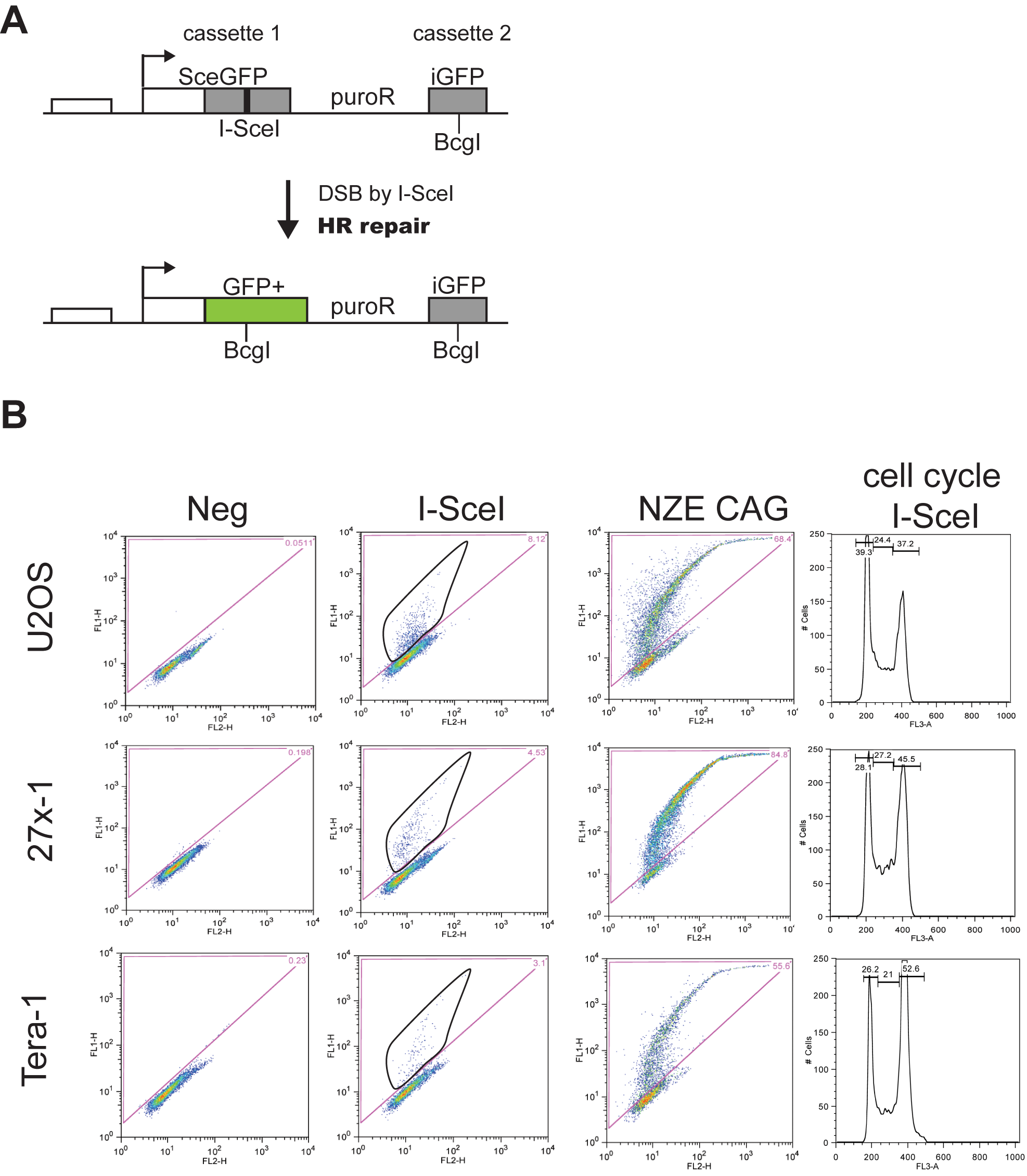

Supplement: Figure S3 — DR-GFP assay. A) Schematic representation of the DR-GFP substrate. The DR-GFP gene is a modified GFP gene in which GFP is modified to SceGFP (cassette 1) so as to contain an ISceI site (incorporated at the BcgI site) and in frame termination codons. Downstream of the SceGFP gene, is an internal GFP fragment (cassette 2). Repair of DR-GFP substrate by homology-direct repair (HR) restore GFP function. B) Representative flow cytometry profile of the indicated cell lines analyzed 48 hs following plasmids transfection. Neg = GFP profile of cells transfected with DR-GFP plasmid plus a control plasmid (pCAGGS). I-SceI = GFP profile of cells transfected with DR-GFP plasmid plus a I-SceI expression plasmid (pCBASce). The circled area indicates the GFP+ cells. NZE CAG = GFP profile of cells transfected with a GFP expressing plasmid (Nze-GFP). The percentage of DR-GFP positive cells was normalized against the percentage of Nze-GFP positive cells (transfection efficiency). (TIF) [file pone.0051563.s003.tif]

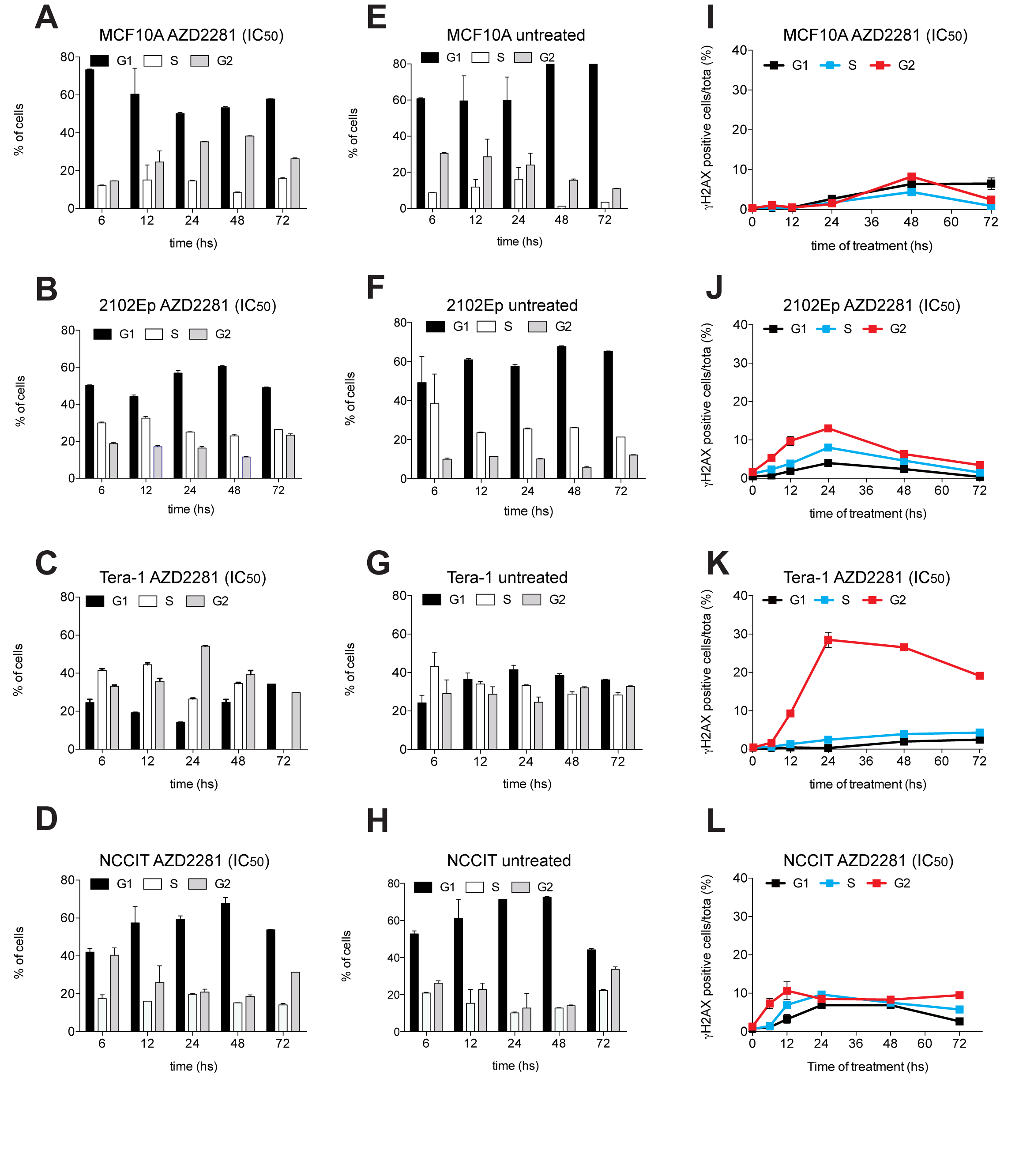

Supplement: Figure S4 — Cell cycle distribution and mean percentage of γH2AX positive cells in G1, S and G2 phases of the cell cycle in exponential phase populations of U2OS and EC cell lines treated (or untreated) with AZD2281. A–D) Cell cycle distribution following AZD2281 treatment. Cells were treated in continuous with the IC50 dose of AZD2281, collected at the indicated time points, and stained with propidium iodide for FACS analysis. E–H) cell cycle distribution of the indicated cell lines in absence of drug treatment. I–L) Cell cycle distribution of γH2AX-positive cells following AZD2281 treatment. The indicated cell lines were treated as described above, collected at the indicated time points, and stained with the anti-γH2AX antibody for FACS analysis. Data are mean value ± s.d. of three independent experiments. (TIF) [file pone.0051563.s004.tif]

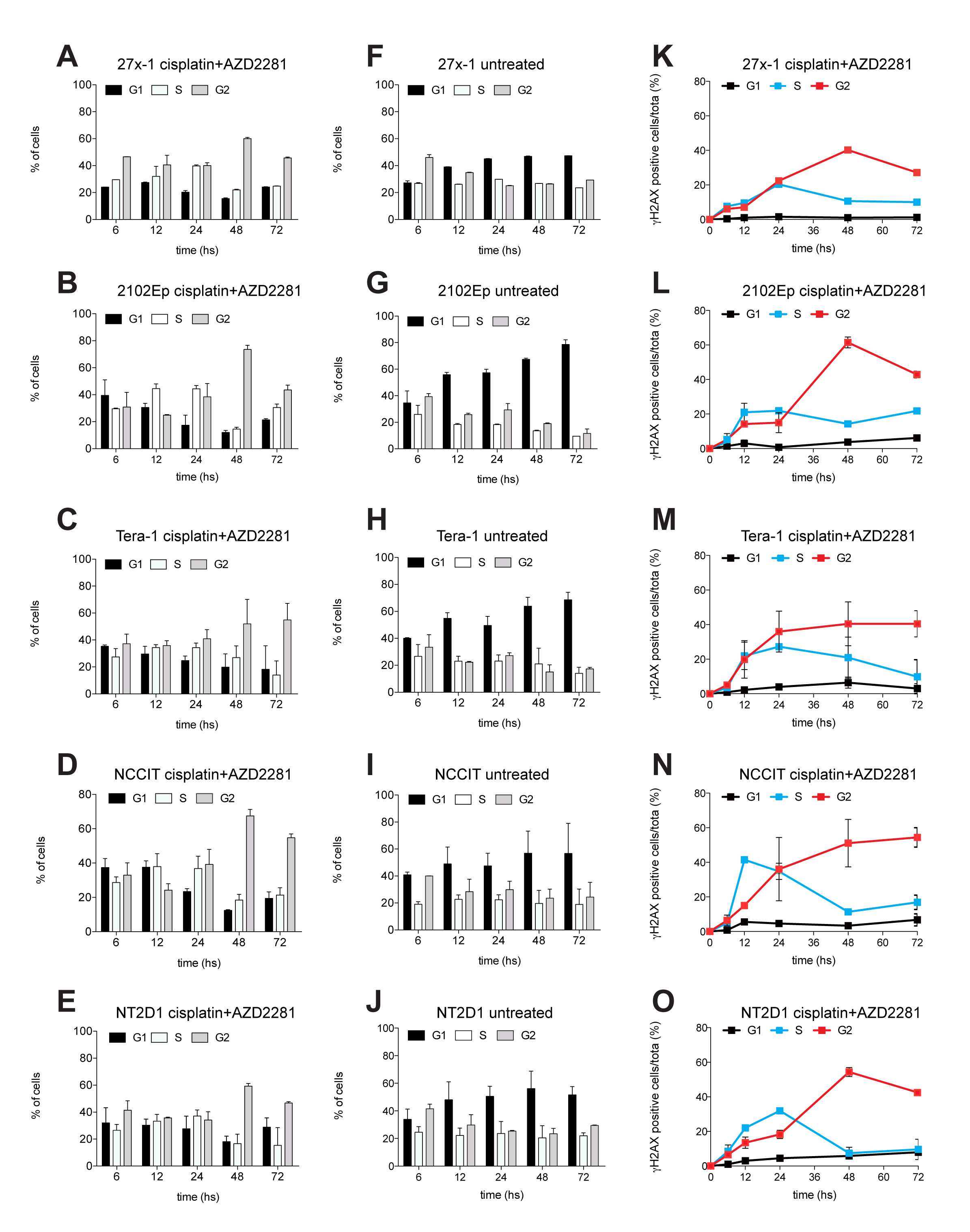

Supplement: Figure S5 — Cell cycle distribution and mean percentage of γH2AX positive cells in G1, S and G2 phases of the cell cycle in exponential phase populations of U2OS and EC cell lines treated (or untreated) with cisplatin/AZD2281 combined therapy. A–E) Cell cycle distribution following cisplatin/AZD2281-combined treatments. Cells were co-treated with cisplatin (at a concentration corresponding to the IC50 of each cell line) and AZD2281 (at a concentration corresponding to the ½ IC50 of each cell line) for 6 hs. At the end of treatment cisplatin was washed out and cells maintained in continuous presence of AZD2281 (½ IC50 dose). Cells were collected at the indicated time points, and stained with propidium iodide for FACS analysis. F–J) cell cycle distribution of the indicated EC cell lines in absence of drug treatment. K–O) Cell cycle distribution of γH2AX-positive cells following cisplatin/AZD2281 combined treatment. The indicated cell lines were treated as described above, collected at the indicated time points, and stained with the anti-γH2AX antibody for FACS analysis. Data are mean value ± s.d. of three independent experiments. (TIF) [file pone.0051563.s005.tif]

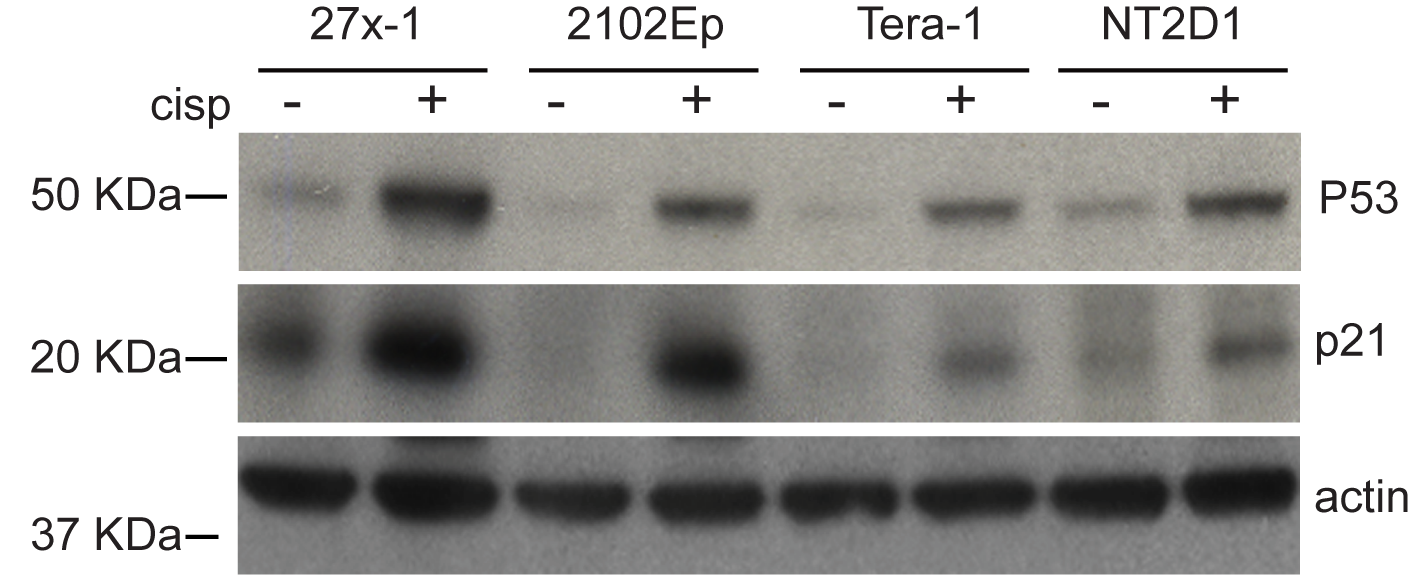

Supplement: Figure S6 — The status of P53 does not predict EC sensitivity to cisplatin. Representative images of a western blotting analysis of p53 and p21 protein levels, following 24 hs treatment with 3.3 µM cisplatin. All cell lines but NCCIT (not shown) are wild-type for TRP53, as shown by the increase of both p53 and p21 proteins level upon cisplatin treatment. Actin was used as loading control. (TIF) [file pone.0051563.s006.tif]

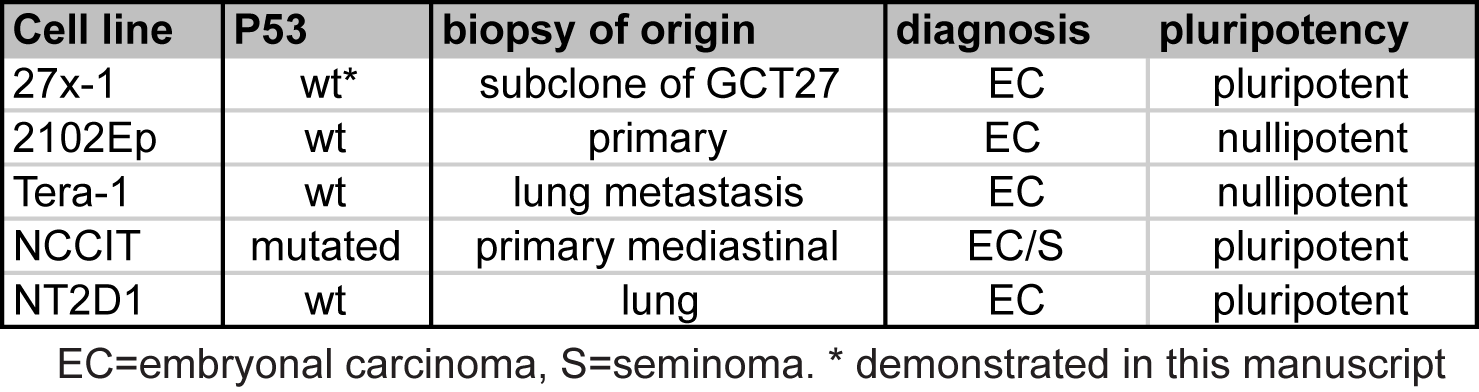

Supplement: Table S1 — Origin of EC cell lines used, with their P53 status. Cell lines are predicted to be either mutant (NCCIT) or wild type (2102Ep, Tera-1, NT2D1) forTRP53. 27x-1 cell line is also wild type for p53, as shown by p53 (and p21) up-regulation upon cisplatin treatment (see Fig. S6). (TIF) [file pone.0051563.s007.tif]
